# Evaluation of the eNutri automated personalised nutrition advice by users and nutrition professionals in the UK

**DOI:** 10.1371/journal.pone.0214931

**Published:** 2019-04-03

**Authors:** Rosalind Fallaize, Rodrigo Zenun Franco, Faustina Hwang, Julie A. Lovegrove

**Affiliations:** 1 Hugh Sinclair Unit of Human Nutrition and Institute for Cardiovascular and Metabolic Research (ICMR), Department of Food and Nutritional Sciences, University of Reading, Whiteknights, Reading, United Kingdom; 2 School of Life and Medical Sciences, University of Hertfordshire, College Lane, Hatfield, United Kingdom; 3 Biomedical Engineering Section, School of Biological Sciences, University of Reading, Reading, United Kingdom; Universitat de Lleida-IRBLLEIDA, SPAIN

## Abstract

Nutrition apps have great potential to support people to improve their diets, but few apps give automated validated personalised nutrition advice. A web app capable of delivering automated personalised food-based nutrition advice (eNutri) was developed. The aims of this study were to i) evaluate and optimise the personalised nutrition report provided by the app and ii) compare the personalised food-based advice with nutrition professionals’ standards to aid validation. A study with nutrition professionals (NP) compared the advice provided by the app against professional Registered Dietitians (RD) (n = 16) and Registered Nutritionists (RN) (n = 16) standards. Each NP received two pre-defined scenarios, comprising an individual’s characteristics and dietary intake based on an analysis of a food frequency questionnaire, along with the nutrition food-based advice that was automatically generated by the app for that individual. NPs were asked to use their professional judgment to consider the scenario, provide their three most relevant recommendations for that individual, then consider the app’s advice and rate their level of agreement via 5-star scales (with 5 as complete agreement). NPs were also asked to comment on the eNutri recommendations, scores generated and overall impression. The mean scores for the appropriateness, relevance and suitability of the eNutri diet messages were 3.5, 3.3 and 3.3 respectively.

## Introduction

Nutrition apps have significant potential to improve health-related food choice. However, our review of popular nutrition-related mobile apps revealed that none of the reviewed apps had a decision engine capable of providing personalised dietary advice [1]. Despite this, a recent three country study (Australia, New Zealand and the UK) showed that nutrition apps were used by the majority of the respondent dietitians (62%, n = 570), as information sources (74%) or for patient self-monitoring (60%) [2]. These data illustrate the high and increasing use of nutrition-related apps by both the public and dietetic professionals. There is a lack of evidence on whether users of nutrition apps are able to understand them (including design, data visualisation, messages) and whether nutrition professionals (NP) agree with the information and advice provided by them.

Two recent systematic reviews found that since 1981 around 30 dietary interventions had been delivered remotely [3,4] using different methods including websites (n = 4) and apps (n = 1) (4). The materials used in these interventions (e.g. printed reports, e-mails, videos, list of SMS messages, decision trees) were rarely described clearly as reported by Warner et al. who stated that out of the 37 eligible trials in their review, 39% reported the intervention material and only 20% described where to find copies of them [3]. To corroborate this, Teasdale et al showed that only five studies, out of a total of 26, did not contain high risk of bias, according to the Cochrane tool. They also reported that the majority of the interventions involved face-to-face interactions before the remote stage [4]. These facts show that the popularity of diet apps are greater than the scientific evidence to support their reliability and effectiveness.

The pan-European Food4Me randomized controlled dietary intervention study investigated whether personalised food-based nutrition advice (based on diet, phenotype or genotype) delivered remotely, motivated participants to make healthy food choices compared with general public health dietary recommendations [5]. Results from this study suggest that online personalised nutrition advice, based on dietary intake (assessed using a validated Food Frequency Questionnaire (FFQ) [6,7] with photographs), was more effective at improving adherence to dietary advice than standard population guidance [8]. The decision tree for tailoring the diet recommendation in the Food4me project is not available in the public domain and validation by independent nutrition professionals, outside of the research team, was not reported.

We have developed a mobile web app capable of delivering automated personalised food-based nutrition advice (eNutri), the source code for which is publicly available [9,10]. Dietary assessment is via the validated Food4Me FFQ [6] with an updated user interface that has been designed for increased usability and the capability to be accessed across multiple commonly-used devices, including tablets and smartphones [11]. A unique feature of the eNutri app is that the dietary advice is derived from adherence to an 11-item modified US Alternative Healthy Eating Index, which we refer to as the m-AHEI. The AHEI was selected for its strong inverse association with CVD [12,13] and markers of adiposity [14], positive association with markers of dietary intake and physical activity and suitability towards Northern-EU countries[14].

During the design process for the advice system, a formative face-to-face study with potential users (n = 20) was conducted to evaluate their understanding of the nutrition report and to inform important changes to the app’s design. Additionally, the app had yet to be compared and validated against professional recommendations (usual care). Assessing whether Registered Dietitians (RD) and Registered Nutritionists (RN) agree with the automated nutrition advice (e.g. its appropriateness, relevance and impact) is important in maximising the success and wider utility of this app. The aim of the present study was to i) evaluate and optimise the personalised nutrition report delivered by the app (with the formative face-to-face study) and ii) compare the personalised food-based advice against health professional standards via a dietary feedback survey comparing the advice provided by the eNutri app against professional RD (n = 16) and RN (n = 16) standards and to collect usual professional recommendations in order to understand where the automated advice can be improved. These studies will help address the need for greater transparency and reproducibility in remotely delivered interventions.

## Materials and methods

Ethical approvals for the formative and NP studies were granted by the Research Ethics Committee of the School of Chemistry, Food and Pharmacy at the University of Reading, UK (Ref No. 04/17 and 11/17, respectively).

### eNutri app

The study relates to the eNutri app [9], in which participants complete an online FFQ and a report with personalised food-based dietary recommendations is automatically generated and presented on the screen. The report comprises an introductory message (e.g. “Hi John, this is your personalised report. The following messages present the most important diet changes recommended for you.”), followed by messages highlighting three dietary changes that the participant is recommended to consider. The messages for the participant are automatically selected by the eNutri app based on the three m-AHEI components with the lowest scores [11].

**Fig 1:**
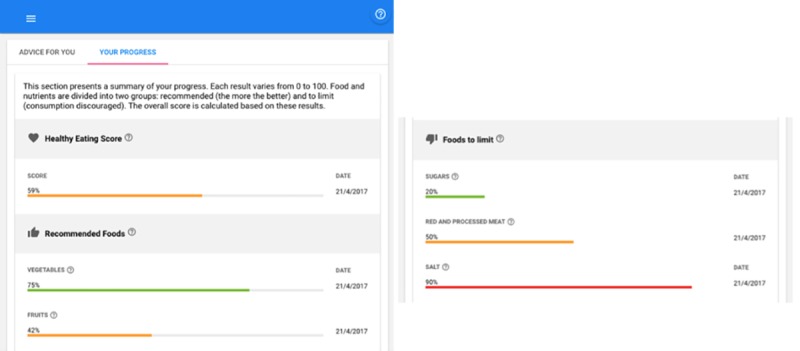
eNutri presenting the ‘Healthy Eating Score’, ‘Recommended foods’ and ‘Foods to limit’. Reprinted from [10] under a CC BY license, with permission from Rodrigo Zenun Franco, 2019.

The report also shows the participant’s m-AHEI score, referred to in the report with a more user-friendly name of “Healthy Eating Score”, presented as a percentage and a coloured bar (Fig 1). The colour of the bar denotes how closely the user complied with the AHEI, with red representing a score in the lowest third, yellow in the middle third, and green in the highest third. Intakes were scored as a percentage of the target as recommended by the m-AHEI. The participant’s score on each of the 11 m-AHEI components are also shown, their scores for ‘recommended foods’ (vegetables, fruits, whole grains, dairy products, nuts & legumes, healthy fats, oily fish) were presented first, followed by their scores for ‘foods to limit’ (sugars, red and processed meat, salt and alcohol). The bars for the ‘Recommended Foods’ used the same traffic light system as the overall “Healthy Eating Score” score. Although the ‘Foods to Limit’ had the same style of bars, the colours were inverted (e.g. red colour if the score exceeded two-thirds of the recommended maximum intake).

**Fig 2:**
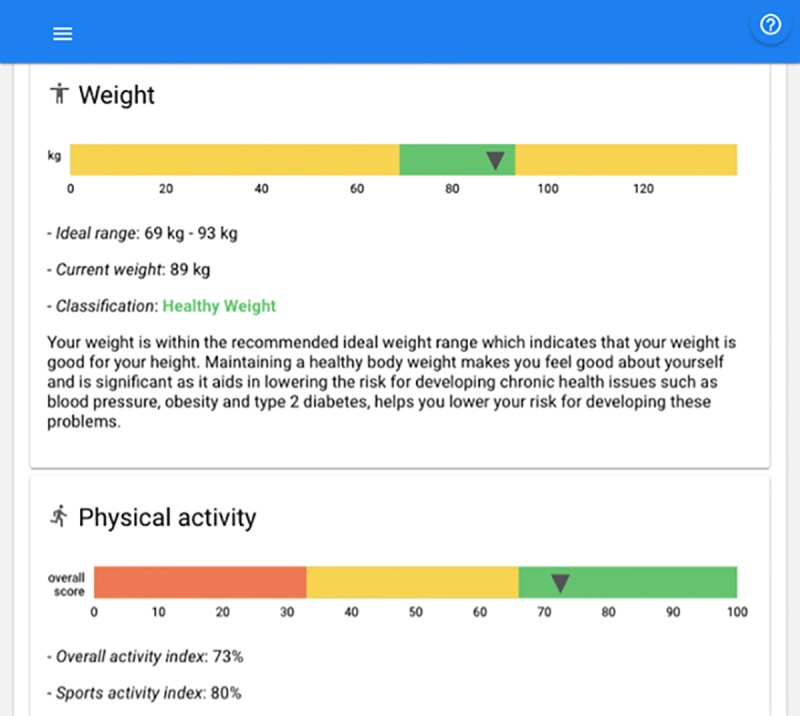
eNutri presenting the weight and physical activity ranges. Reprinted from [10] under a CC BY license, with permission from Rodrigo Zenun Franco, 2019.

A user could see a weight range bar and recommendations based on their body mass index (BMI). The report did not present the BMI explicitly, but used the healthy BMI range (18.5-25kg/m^2^) to calculate the healthy weight range for the user’s height, and this range was presented in green. An arrow on the bar indicated the user’s current weight [11]. The physical activity levels are defined based on the Baecke Questionnaire [15], which is a questionnaire of habitual physical activity. It has been validated and found to be repeatable [16] (Fig 2).

### Formative study

For a fuller understanding of the study described in the next sections involving evaluation of the eNutri app by nutrition professionals, this section describes some of the background to and rationale behind the design of the personalised diet report. Using an initial version of the app described in [11], a formative study with 20 participants, without nutrition expertise, was conducted. The participants’ characteristics of the formative study have been described by Franco and colleagues in a publication that presented the evaluation of the FFQ (9) but not the evaluation of the nutrition report. Here, we report the latter which was evaluated via a semi-structured face-to-face interview (S1 File) and addressed the first aim of the study to i) evaluate and optimise the personalised nutrition report and app delivery. The interview included questions regarding participants’ understanding of the content and terms used in the diet messages, and also the visual representation of the status bars [11]. The interviews also explored questions related to the perceived effectiveness of the report and ideas for potential improvements and new features for the system. An interview protocol, available as supporting information (S1 File), was used by the researcher. Positive comments, negative comments and suggestions were grouped and counted. The results of this formative study and how they influenced the design of the nutrition report are summarized in the following paragraphs.

The most frequently displayed components (according to the participants’ FFQ results) were “nuts and legumes” (27%), “red and processed meat” (27%) and “salt” (15%). The 20 participants indicated that they understood all the content and terms used in the advice messages. Two participants clicked on the small superscript help icons (Fig 1). Only one participant clicked on the “Your Progress” tab (Fig 1), which was the second option on the menu tab, while for the remaining 19 participants, the researcher had to point out the second tab.

The 2 and 3-colour progress bars used for weight and physical activity [11] received satisfactory responses, confirming participants’ understanding of these visual representations. One participant stated clearly that “I am thinking as a traffic light system”. Regarding the single colour status bars used for the food-based scores (Fig 1), the participants provided very good explanations for the traffic light representation for both categories (e.g. red colour in ‘Recommended Foods’ means to eat more and in ‘Foods to Limit’ means to decrease consumption). When asked, they were also able to compare the components (e.g. the ‘Vegetables intake’ is better than the ‘Fruits intake’ in Fig 1) for both categories, although they were not sure of the meaning when the score was 100% (i.e. full bar). One participant stated: “I understand the message, but I am not sure about the percent of what”. For the ‘Recommended Foods’, responses including “the higher, the better”, “I am eating 75% of what I should” were given, but some clarified that they were not sure if they should be eating exactly 100% and not more than that. For the ‘Foods to Limit’, the responses were much less satisfactory compared with responses for the ‘Recommended Foods’, in the context of what the bars were designed to represent, and it seemed that understanding of the messages were based on the traffic light colours only. In other words, the status bar was not increasing the user’s understanding.

**Fig 3:**
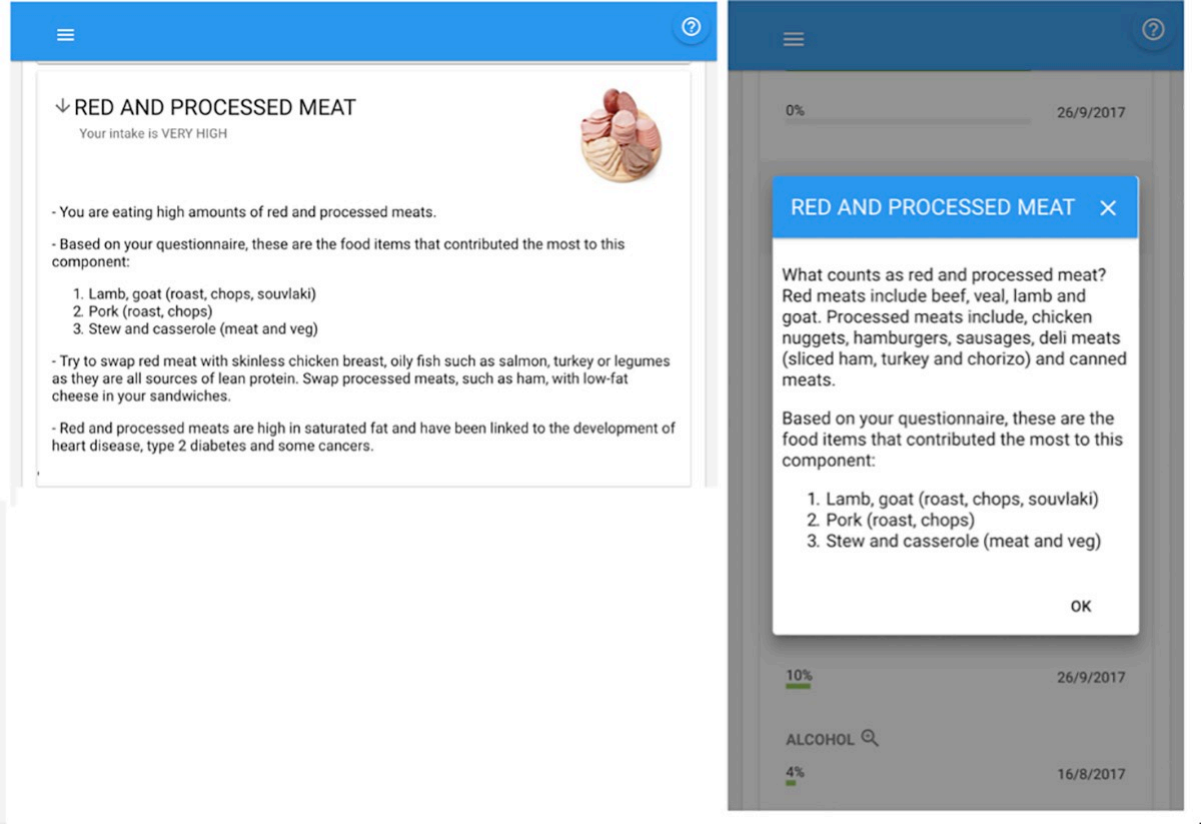
Advice on the biggest contributors to a component as main advice and component details. Reprinted from [10] under a CC BY license, with permission from Rodrigo Zenun Franco, 2019.

Based on the participants’ feedback, a number of improvements were applied to the eNutri report based on the ‘main suggestions’ of the non-professional group and taken forward into the study with the NP. These are summarised below:

- The two tabs (“Advice for you” and “Your progress”) of the report (Fig 1) were merged into a single tab and the progress report was placed below the advice messages;
- Clarification of which individual food items had the greatest contribution to the recommended foods or foods to limit (e.g. which items were the top contributors to “red and processed meat”) (Fig 3);
- Explanatory subheadings of the two component groups were included in parenthesis: Recommended Foods (The higher the better) and Foods to Limit (The lower the better).
- Each main message block (Fig 3) was revised to a standard format: the first paragraph focused on the current status of the specific component; the second provided advice (i.e. “call to action”) and the final paragraph described the health benefit potentially obtained from that specific behaviour change.

### Participants

The revised design of the reports were evaluated with RD and RN with more than 2 years experience of providing individual dietary advice. RDs were recruited via the 'Freelance/Private Practice Dietitian's' Facebook Group, which included 945 members at the time of the recruitment post. A total of 28 RD responded to the request for study volunteers within 12 hours of the post, after which time the researcher [RF] advised the group that the recruitment target had been met. RD eligibility was verified according to participants’ Health and Care Professions Council (HCPC) registration number (http://www.hcpc-uk.co.uk).

RN were recruited from the Association for Nutrition (AfN) website (www.associationfornutrition.org) via the 'search the register' function. Individuals registered as a Nutritionist on the AfN voluntary register who declared that they 'accepted clients' were contacted (n = 85). The study was also advertised on the researcher’s Twitter account and re-tweeted by the AfN. In total, 21 RN responded to the email and 2 to the tweet. For both RD and RN, the first 16 participants who responded were included in the study.

### Study design

Recruited NP (n = 32) were invited to complete an online survey evaluating the eNutri app, via JotForm (jotform.com, San Francisco, CA). Following online screening and consent, each participant was sent two pre-defined scenarios, comprising dietary intake analysis of FFQ data (portion, frequency and weight per food item, and energy and nutrient intake per day) and individual characteristics (age, sex, weight, height and BMI). A total of 16 scenarios were designed to mimic outputs from Nutritics Professional Analysis Software (Nutritics Limited, Dublin, Ireland) (S2 File) and included the Dietary Reference Values (DRVs) provided by the Committee on Medical Aspects of Food and Nutrition Policy (COMA) and the Scientific Advisory Committee for Nutrition (SACN) in the UK (year 2015) [17]. NPs were randomly assigned two of the 16 scenarios and asked to consider these scenarios using their professional judgment and provide the three most relevant nutrition recommendations, via JotForm (Q1). Participants were asked to provide responses via audio recording or free-text boxes.

Following this stage, NPs were asked to access the nutrition advice (i.e. not including the weight and physical activity blocks shown in Fig 2) generated by eNutri for each scenario (via a password-protected link) and rate their level of agreement, considering its appropriateness, relevance and suitability, via 5-star scales (5 denoted complete agreement and 1 no agreement). NPs were also asked to comment (via audio or free-text) on the eNutri recommendations (Q2), scores generated (Q3) and overall impression (Q4), according to each scenario presented. Finally, participants were asked to provide any final comments or feedback on the eNutri app, irrespective of the individual scenarios evaluated.

#### Scenarios

Each of the 16 scenarios (where a scenario comprised a FFQ completed by an individual) was independently evaluated by four NP (2 RD and 2 RN). With this approach, each scenario received recommendations and evaluations by 4 different NPs. The scenarios were designed to represent a diversity of diets and individuals. The diet variable defined was the m-AHEI with selection of individuals also based on sex, age and BMI. These four variables at two levels each (m-AHEI: <40 or >60, sex: M or F, age: <40 or >40 and BMI: Ideal or Overweight) were used to generate 16 distinct combinations as shown in Table 1. The FFQ scenarios (n = 16) were drawn from a pool of FFQ responses from actual users, and so have good ecological validity. In total, 64 analyses were undertaken (16 scenarios x 4 analyses per scenario).

**Table 1:** Characteristics of subjects presented to nutrition professionals according to scenario.

Scenario	Sex^a^	Age	BMI^b^	m-AHEI^c^
**1**	M	<40	Ideal	<40
**2**	M	<40	Ideal	>60
**3**	M	<40	Overweight	<40
**4**	M	<40	Overweight	>60
**5**	M	>40	Ideal	<40
**6**	M	>40	Ideal	>60
**7**	M	>40	Overweight	<40
**8**	M	>40	Overweight	>60
**9**	F	<40	Ideal	<40
**10**	F	<40	Ideal	>60
**11**	F	<40	Overweight	<40
**12**	F	<40	Overweight	>60
**13**	F	>40	Ideal	<40
**14**	F	>40	Ideal	>60
**15**	F	>40	Overweight	<40
**16**	F	>40	Overweight	>60

^a^ Male, M; female, F

^b^ Ideal, BMI 18.5–24.9 kg/m^2^; overweight, BMI >25kg/m^2^

^c^ modified alternate healthy eating index

The characteristics presented in Table 1 and their associated FFQs were entered into the app database in order to simulate the nutrition reports for the scenarios (S2 File). The NPs were able to browse the reports online and their screenshots are presented in S3 File.

### Data analysis

#### Quantitative analysis

Participant characteristics (age and years since graduation) were presented as means ± standard deviations according to type of nutrition profession and compared using an independent samples t-test.

#### Qualitative analysis

Targets for diet change recommended by the NP were collated by scenario and compared with the changes targeted by the eNutri app. Free-text comments were analysed using an inductive thematic approach [18], similar to that described by Cunningham & Wells [19]. Responses were analysed according to question and thus, divided into four data sets (Q1, NP nutritional suggestions; Q2, NP comments on eNutri ratings; Q3, NP evaluation of eNutri scores; Q4, overall impression and final comments). NP suggestions (data set 1) were analysed to identify themes in relation to provision of nutritional advice via the following steps: familiarisation, coding, identification of subthemes and overarching themes, and interpretation. All data analyses were conducted by the primary author (RF) and independently verified by the second author (RZF).

Following familiarization, responses for data sets Q2-Q4 were broadly categorized into (1) positive (e.g. beneficial aspects), (2) neutral and (3) negative comments (e.g. aspects to improve upon) prior to further analysis. Comments were then coded, and themes/subthemes identified. Codes with similar content and meaning, for example ‘personal preference’, ‘cooking abilities’, ‘adverse reactions’ and ‘body composition’ were amalgamated into a single theme: ‘consideration of wider context’. Where topics/ generated themes resulted in both positive and negative feedback (e.g. aspects of the app praised by some NP, and criticised by others), this was identified and included in the results. NP responses to Q1-Q4 could contribute to multiple themes. Quotes are presented according to anonymised identification codes.

## Results

### Participant characteristics

Participant characteristics according to profession (RD, n = 16; RN, n = 16) are shown in Table 2. 90% of NPs were female. The mean age was 37 years (SD 10) and reported years since graduation was 10.9 (SD 8.2). No significant differences were observed between the professions.

**Table 2:** Participant characteristics according to profession (RD, n = 16; RN, n = 16)^a^.

	All (n = 32)	RD (n = 16)	RN (n = 16)	*P* Value^b^
**Sex (M/F)**	3/29	1/15	2/14	n/a
**Age (years)**^**c**^	37 ± 10	36 ± 10	39 ± 9	0.346
**Years since graduation**^**d**^	10.8 ± 8.1	8.4 ± 6.5	14.2 ± 8.9	0.077

Data are means ± standard deviation

^a^ Registered Dietitian, RD; Registered Nutritionist, RN

^b^ Data analysed using independent samples t-test

^c^ Data not provided by n = 1 RD and n = 3 RN

^d^ Data not provided by n = 2 RN

### Professional recommendations

**Fig 4:**
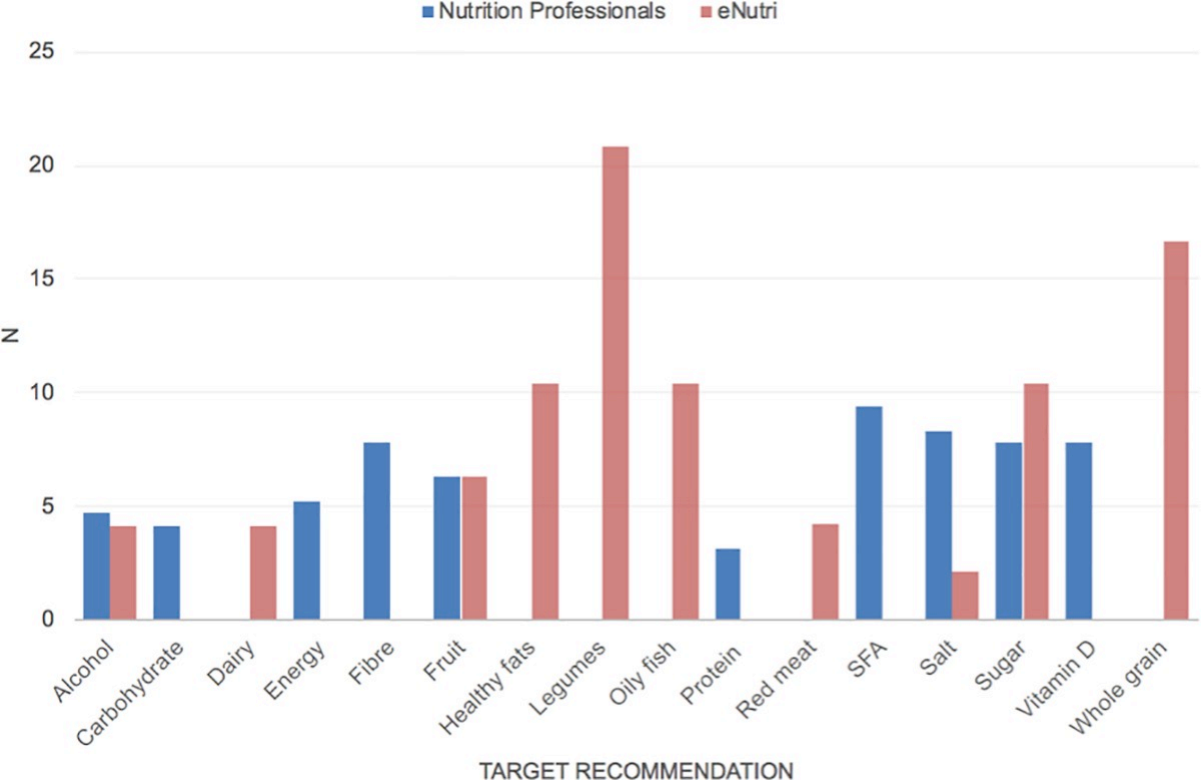
Number of times each target recommendation was selected by nutrition professionals and by the eNutri app for all scenarios combined.

The option to provide recommendations via audio recording was not used by any NP. The primary nutritional targets (n = 3) listed by NPs following evaluation of the scenarios are shown in Fig 4 and detailed in the S1 Table; a total of twelve targets are listed per scenario (4 NP x 3 recommendations), although this analysis does not illustrate the agreement at the level of the individual scenarios. In total, 43 distinct targets were selected, which included nutrients (e.g. fibre), food items (e.g. red meat), eating occasions (e.g. breakfast) or ‘ratios’ of different nutrients/composition of macronutrients in the diet (e.g. ratio of saturated to unsaturated fats). Targets were not set on five occasions (scenarios 8, 14 and 16), whereby the NPs described needing further information in order to ascribe a recommendation. For scenario 2, one NP stated that the individual’s diet was “ok”/required no improvement. The greatest agreement in targets selected by NP, according to scenario, (selections per scenario > 3) was observed for energy, macronutrient targets (e.g. saturated fatty acids (SFA), fibre, alcohol) and fruit. Less agreement was seen for micronutrients (e.g. phosphorus, iron, vitamin A, selenium), which NP selected less frequently and accounted for < 10% of the total targets. The top five items targeted by NP, according to overall frequency, were SFA (9.4%), salt (8.3%), fibre (7.8%), sugar (7.8%) and vitamin D (7.8%).

In the majority of cases a nutrient was the first target mentioned by the NP, although this was frequently followed up with food-based advice; for example: “Increase intake of dietary fibre by increasing whole grain food instead of white bread, non wholegrain cereals” (RN16) and “reduce sugars: reducing intake of sweet biscuits, chocolate, rich cakes, buns, muffins, pastries (…)” (RD04). In some cases, NPs also mentioned the reason and associated health benefits of implementing the suggested changes; for example: “It is important that you start your day with a nutrient rich breakfast as this will not only help to keep your energy levels steady, but it will also support cognitive function helping to keep you focused and alert throughout the morning” (RN05) and “Reducing his intake of alcohol could help to reduce his weight” (RD03). Some NP also listed likely improvements to other nutrients, in addition to that targeted, for example: “try to include 2 portions of oily fish per week (…) This would also boost vitamin D and iron intake” (RD15). Alternatively, some described the consequences of current intake/habit: “Deficiency of vitamin A can cause fatigue, increase risk of infection, poor vision and dryness of the eyes, skin and hair” (RN06).

Nutrition recommendations were typically framed according to “increase”/”choose”/”consume more” or “reduce”/”decrease”/”limit”/”stop”/”cut down”, and less frequently as substitutions or food-swaps; for example: “Swap jams/marmalade/chocolate and nut spreads for low-fat cheese spreads” (RN10) and “(…) reducing intake of biscuits, chocolate and fizzy drinks. In place of these try fruit and natural yoghurt for puddings (…)” (RD02).

Overall, the detail provided by the NPs for each recommendation varied (range 12–176 words), with the greatest quantity of text provided for a “calorie deficit” recommendation; in this case, the NP described the weight loss target, energy requirements and deficit, and a list of food based swaps and portion size recommendations to facilitate a calorie reduction (e.g. “grill fish rather than fry”) (RN10). NPs referred to individuals’ characteristics, and particularly BMI for overweight cases, where relevant; recommendations to reduce energy were also more frequent in scenarios with overweight individuals (3–4, 7–8, 11–12, 15–16) (see Table 1). Females of childbearing age (“females of birthing age”, “(…) she may be wishing to conceive”) were also highlighted in relation to recommendations to increase iodine and iron (scenario 9), folate (scenario 12) and reduce protein by “(…) reducing number of oily fish portions per week (no more than 2 per week recommended” (scenario 10, RN10).

Vitamin D was targeted by at least one NP in the majority of scenarios; follow-up recommendations included the consumption of vitamin D rich food/fortified products: “Increase intake of vitamin D foods e.g. dairy produce, oily fish, eggs, fortified products” (RD16) or supplementation during the autumn/winter months; for example “Depending on time spent outside in the summer, take a vitamin D supplement to ensure (…) intake averages out to 10ug/day” (RN07).

### Evaluation of the eNutri feedback

The diet targets automatically selected by the eNutri app and the professional evaluation of the advice generated, according to each scenario, are shown in Table 3. The most frequently selected items, according to overall frequency, were legumes (20.8%), whole grain (16.7%), and red meat, oily fish, healthy fats and sugar (all 10.4%). The mean scores for the appropriateness, relevance and suitability of the eNutri diet messages were 3.5, 3.3 and 3.3 respectively (maximum score, 5). Two scenarios (1 and 3) scored > 4 on all three aspects and the lowest mean scores (2.5) were observed for scenarios 15 and 16, which were both overweight females, aged > 40 years.

**Table 3:** Professional evaluation of eNutri automated personalized nutrition advice according to scenarios (n = 4 responses per scenario).

N	eNutri targets			Appropriateness	Relevance	Suitability
	1	2	3	(max = 5)	(max = 5)	(max = 5)
**1**	Red meat	Oily fish	Fruit	4.0 ± 1.2	4.0 ± 1.2	4.0 ± 1.2
**2**	Legume	Salt	Healthy fat	3.5 ± 1.1	3.5 ± 1.1	3.3 ± 1.1
**3**	Alcohol	Red meat	Oily fish	4.8 ± 0.4	4.0 ± 1.0	4.0 ± 1.0
**4**	Red meat	Fruit	Legume	3.8 ± 0.4	4.3 ± 0.4	3.5 ± 0.5
**5**	Red meat	Legume	Whole grain	4.3 ± 0.4	3.8 ± 0.8	4.0 ± 0.7
**6**	Dairy	Whole grain	Oily fish	3.3 ± 0.8	2.8 ± 1.1	2.8 ± 1.1
**7**	Whole grain	Red meat	Oily fish	3.8 ± 0.4	3.5 ± 0.9	4.0 ± 1.2
**8**	Legume	Red meat	Whole grain	3.0 ± 1.2	3.0 ± 1.2	3.0 ± 1.2
**9**	Whole grain	Legume	Sugar	3.3 ± 0.4	2.8 ± 1.1	3.3 ± 0.4
**10**	Legume	Sugar	Healthy fats	4.0 ± 0.7	4.0 ± 0.7	3.8 ± 1.3
**11**	Sugar	Red meat	Legume	3.8 ± 0.8	3.0 ± 1.4	3.0 ± 1.4
**12**	Healthy fat	Fruit	Dairy	3.3 ± 1.3	3.3 ± 0.8	3.0 ± 1.0
**13**	Alcohol	Whole grain	Legume	3.5 ± 0.5	3.5 ± 1.1	3.3 ± 0.8
**14**	Whole grain	Healthy fat	Sugar	3.5 ± 0.9	3.0 ± 0.7	3.5 ± 0.9
**15**	Whole grain	Legume	Oily fish	2.5 ± 1.5	2.5 ± 1.5	2.5 ± 1.5
**16**	Legume	Healthy fat	Sugar	2.5 ± 0.5	2.5 ± 0.5	2.5 ± 0.5
**Average**	-	**-**	**-**	**3.5 ± 1.0**	**3.3 ± 1.2**	**3.3 ± 1.2**

#### Recommendations

The NPs comments regarding the eNutri recommendations predominantly related to whether the NP agreed with the three food components selected by the eNutri app. Positive quotes included: “All advice given was very appropriate” (RD16), “I definitely agree with the recommendations provided and would say they are more suitable than the ones I have provided! Great practical advice provided in the report” (RD15) and “Good advice relevant to diet” (RN03). However, considering the differences between the NP and eNutri recommendations (see Tables 3 and 4), preferential targets were frequently mentioned: “Give good information regarding the topics, although I feel some of the areas chosen by the software were not the most important aspect to change in terms of the clients diet” (RN08). Particular components perceived to be important and omitted from the eNutri app advice included saturated fat, vitamin D and micronutrients. NP also queried the prioritisation of components such as whole grain when dietary fibre was adequate; and dairy when calcium and protein were sufficient.

**Table 4:** Quotes identifying positive and negative aspects of the eNutri automated personalized nutrition app scoring system identified by nutrition professionals.

Positive aspects eNutri scoring	Negative aspects eNutri scoring
“I like the colour codes of red, amber and green” (RD16)	“I found the scores to be unclear and therefore unhelpful” (RD11)
“I like that you can click on the + button for additional information” (RN16)	“It could appear very disheartening to be red” (RD03)
“The visual scale would probably help to focus a client” (RD03)	“(…) it is hard to interpret these. 100% of what?” (RN11)
“Good scale, makes it easy to see where needs improvements” (RN03)	“Too many negative scores less likely to bring about change” (RD14)
“The scores are very clear and give excellent guidance for changes to be made” (RN14)	“I think having red and processed meat in the same category is a bit misleading (…) these should be separated” (RD05)
“Good scoring to highlight areas of change” (RD07)	“The scores are very harsh????” (RD08)
“Little room for misinterpretation by the person who would be looking at this information” (RD09)	“It felt too overwhelming (…) I think that was due to the higher the better/ lower the better system” (RN04)
“Appears to reflect the individuals food choices” (RN05)	“Dislike healthy eating score because dietary intake is personal and cannot follow gov guidelines for all” (RD14)
“Good way to rate the diet” (RD07)	

Themes largely mapped to those identified in the analysis of NP recommendations, including message framing, client context (e.g. allergies) and reason for implementing suggested recommendation. There were contradictory opinions regarding the quantity of information presented to the individual: “recommendations are fine on but worrying that it is limited to 3?” (RN16) versus “It felt too overwhelming (…)” (RN04). NPs commented that client context, including allergies, intolerances, religious beliefs and food preferences, should be considered further to increase the ‘personalisation’ of the recommendations. For example, it was noted that in certain scenarios, individuals reported 0g of red meat, fish and dairy intake, yet advice included the consumption of more oily fish; thus, it was highlighted by a NP the “Need to discuss alternatives to oily fish for vegetarians or none [sic] fish eaters” (RD14). A NP also commented that: “It is not a tool that would help a professional especially if they were looking to help with health issues as it does not look at the individual—it is based on general dietary advice but what happens if that person has an allergy […]” (RN02).

The inclusion of descriptions relating to the benefit of implementing a dietary change were perceived as positive: “I liked that the advice included the reasoning for the recommendations, and it was great that it mentioned preventing the non-communicable diseases”, although it was suggested that more relevant conditions could be mentioned according to the specific case: “The participant was a 25 year old male, who may not yet be concerned about Heart Disease etc. It may have been more appropriate to also mention about other benefits of the recommended foods, such as increasing satiety.” (RN04). Message framing was also praised: “(…) the positive reinforcement preceding the recommendation is an excellent way to introduce this change.” (RD12).

#### Scores

Quotes relating to NP appraisal of the eNutri scoring system are summarised in Table 5. In general, NP described ‘agreement’ with the scoring system (i.e. components and scales) and scores in the context of the scenarios’ nutritional analysis, for example: “Appears to reflect the individuals food choices” (RN05), “I felt the scores were all accurate and suitable for this lady” (RD16) and “the healthy eating score is reasonable” (RN09). Although, a few NP stated, “The healthy eating score is very low; it should be higher” (RN09) and “82% seems a bit low given that the diet is pretty good” (RN07). Perceived benefits of the scoring system included the presentation of the data according to the traffic light system and focusing the client to an area of diet that needs improving.

**Table 5:** Positive aspects and areas for improvement to the eNutri automated personalized nutrition advice identified by nutrition professionals.

Positive aspects eNutri	Suggested improvements
Focus on food items vs. nutrients	Greater focus on energy balance for overweight individuals
Clear presentation	Inclusion of advice on vitamin D supplementation
User friendly	Consideration of wider context (e.g. ethnicity, lifestyle issues)
Easy to read messages	Information on how to achieve ‘100%’ for a component
Positive reinforcement of good habits	Include information on food quality and nutrient density
Visual presentation and use of traffic light system	Include a message on the overall diet/m-AHEI score
Considered foods to add in addition to those to remove	Describe the maximum values for each component (e.g. 5 portions of vegetables)
Practical recommendations	Visual representation of data (e.g. pie/bar chart)
Focus on aspects of diet	Display/ indicate how scores will change if diet advice followed
Use of ‘food swaps’	Provide links to recipes
	Sources of additional information (e.g. NHS website)
	Inclusion of a print function

However, concerns were raised regarding the benefit of a scoring approach: “I’m not convinced about the scoring system. It could appeal to those with competitive edge, but many of my clients would not be interested (…)” (RN04), including whether it would be understood: “Scores are useful but may be confusing to someone without a nutritional background” (RD16) or useful: “most people know if their diet is not healthy–the difficulty is how to change (…) scoring them could just be another indicator that they are not capable of getting their life in order” (RN02). It was also noted that a predominance of ‘red’/’negative’ scores might be demotivating.

Within the comments relating to scores, NP also highlighted particular components that would benefit from refinement; for example, the combination of red and processed meat in the context of achieving sufficient iron intake was raised: “(it’s) risky to be advising intakes (of red and processed meat) should be ‘as low as possible’ when female, and clients intakes are low” (RN15). The absence of a component on total and saturated fat was also raised and a NP commented that feedback “Should consider fruit carbohydrate sugars in the sugar recommendation or provide comment” (RD14).

In general, opinions on the scoring system differed, as illustrated in Table 4, however a consistent theme was the desire for greater understanding of the scoring system in terms of an absolute amount (e.g. users intake marked as 2 portions/160g of vegetables), as opposed to percentage of an unknown maximum.

### Overall impressions and suggestions

Positive aspects and areas for improvement to the eNutri app identified by NP are summarised in Table 5. Positive comments were generally shorter in length compared with negative comments/ recommended improvements. NP were positive about the ‘food-based’ approach used in the eNutri app: “(…) It was useful that individual food groups were recommended, rather that macronutrients as this may appeal to individuals who are less educated in nutrition” (RN04); although, several NPs commented on the need for specific advice on vitamin D intake and supplementation. One NP also perceived a lack of information about saturated fat as “a big gap” (RD10). The consideration of ‘foods to add’ in addition to ‘foods to limit/cut out’ was also considered positive, as was the framing of foods that should be eaten less frequently (i.e. foods to limit): “(…) I also like the fact that it describes food to “limit” rather that totally avoid which is a healthier message to spread” (RN04).

Feedback relating to the visual presentation of the data (e.g. key messages, scores, use of traffic light system) was largely positive, for example: “the use of a scoring system is a helpful motivating measure for participants to make recommended changes” (RD12), “quite impressed with the report generated” (RD13), “Good to provide swap ideas” (RD01) and “really practical recommendations highlighting several important changes which could be made” (RD03). However, contrary opinions were also noted in relation to specific scenarios and, when comparing reports, a NP commented: “I am less happy with these recommendations. They are very difficult to put into practice” (RN11). In addition, NP raised concerns with the intake of certain foods, such as nuts and low-fat dairy products, being described in terms of ‘the more the better’.

Of particular note was feedback relating to overweight participants, and NP who reviewed these scenarios commented on the need for a greater focus on energy balance, nutrient density, motivation and weight loss: “Does not consider the need to lose weight and have a healthy BMI” (RD01); “too basic and general for someone who clearly needs nutritional help–they are overweight so the chances are they know diets inside out already” (RN02); “I don’t think the advice was correct for this patient as it did not take into account the fact she was overweight” (RD05). In relation to this, NP suggested that messages should include content on energy reduction and portion sizes.

Overall, NP considered that the system and associated advice was ‘basic’, provided “Good advice for general healthy eating feedback” (RD07) and was “(…) ok for someone who just wants to see where his diet needs improving (…)” (RN02). However, the advice was also described as: “too basic and generalised” (RN02) and that the advice “could be more personalised e.g. inclusion of (fibre-containing) nuts/oats in the participants' smoothies which would be in line with their existing intake and thereby making it more meaningful and easier to incorporate” (RD12) and “still far from a personalised advice that takes into consideration the preferences, adverse reactions to foods, body composition, etc.” (RN09). Whilst it was acknowledged that an app might not be capable of providing the same ‘level’ of personalisation as a NP, several areas for improvement were noted, including: relating advice to a user’s actual intake, providing links to recipes, ability for the user to interact with recommendations (e.g. select those they feel most able to implement) and including a message on the overall diet.

NP also commented on the need to consider the wider ‘context of the client’ and lack of ‘big picture’ when providing dietary advice, for example: “Need to understand background such as cholesterol and blood pressure” (RN02), “consideration of body composition” (RN09), “consideration of personal preferences/ cooking abilities” (RD11), noting that the system “does not take into context wider health and lifestyle issues” (RN15). A suggested improvement was that wider health issues be considered within the app: “I think it would be good to get an indicator of levels of activity and lifestyle choices e.g. smoking as you have mentioned alcohol but also I think as HCP we should also be encouraging healthier lifestyles in regards to alcohol, smoking and exercise” (RD09).

Specific improvements relating to the scores were mentioned, as there was some confusion regarding the percentage system; NP suggested that the absolute amount corresponding to the maximum score be provided for each component (e.g. a score of 100% for vegetables equals 3 portions per day) and that potential improvements in scores (i.e. if changes were made) might be visually presented to the user to enhance motivation. Contradictory comments were made in relation to the framing of diet messages, which some NP commented were ‘slightly judgemental’/’harsh’ and others described as ‘quite positive’, for example “The inclusion of positive reinforcement with words such as 'Good Job' also lends a personal, positive touch” (RD12).

## Discussion

The interest in diet apps from both the public [1] and NP [2] reinforces the importance of studies that evaluate the suitability of dietary advice that is delivered by the apps. The participants of the formative and the NP studies confirmed that the diet messages (texts) were clear and highlighted the benefit of using a traffic light system in the data visualisation. They reported good understanding of the diet messages, which was in a large part due to the clear food-based recommendations, instead of advice that focused on nutrients, which are difficult to translate to dietary changes. Furthermore, practical advice on food swapping was given that facilitated easy application of the personalised advice to everyday food choice. The evaluation of appropriateness, relevance and suitability of the eNutri advice by the NP indicate a good acceptance by this group.

The NP provided recommendations regarding inclusion of advice on dietary energy in 10 out of the 64 recommendations. The eNutri app does not provide recommendations about energy intake explicitly but does consider dietary energy in the calculation of two m-AHEI components (Free Sugars and PUFA). Two of the 11 AHEI components take sex into account in the score calculation [12]. Age and BMI are not usually embedded within indexes of overall diet quality [20] and the fact that eNutri was not tailoring the diet advice to encourage weight loss for the overweight participants was emphasized by some NP. In comparison, popular nutrition-related apps focus particularly on calorie counting to assist weight loss or maintenance [1]. Specific advice relating to weight loss or gain, where appropriate, could be considered for inclusion in future versions of the eNutri app, which may further personalise the advice to motivate dietary changes with an ideal body weight as a target.

The scenarios created from representative users of this app scored around 50 out of 100 in the m-AHEI (i.e. displayed in an amber colour and a bar extending to the middle of the scale), which supports data from other studies using similar indexes [8,12], however this may endorse the concerns raised by the NP that this diet score may be “harsh” and discouraging. There were some useful suggestions received from the participants on the formative and NP studies which could be incorporated into the eNutri and/or similar personalised nutrition apps. Among these are the availability of recipes that include the specific foods that have been advised, incorporation of data related to energy balance for overweight or underweight users, links to additional sources of information (e.g. on health and wellbeing) and the consideration of other factors such as ethnicity and lifestyle issues that can inform further personalisation.

Comments on the lack of vitamin D advice were given in 25% of the analyses. This may have been motivated by the recent attention on vitamin D, particularly with the new SACN recommendations of a daily intake of 10μg of vitamin D for all adults [21] and recognition of the low vitamin D status in European populations [22]. It was therefore suggested that personalised diet advice should consider vitamin D in these populations. To enable this, the dietary intake assessment method (e.g. FFQ) could be adapted to better capture vitamin D intake by collecting information on vitamin supplementation, which could be used to facilitate personalised vitamin D advice.

There was a diversity of feedback targets (i.e. average of 8 different targets, which ranged between 5 and 9 targets per scenario). This diversity of targets creates a challenge for developing a system for generating automated recommendations. In other words, it would require a substantial quantity of scenarios and NP feedback to train a decision engine based on this information. Despite some limitations of using diet scores as the foundation of the decision engine, without taking BMI and age into account, this approach provided a more quantitative tailoring of the online diet advice, supported by scientific evidence, which was related to the diet score selected. There is a need to design and evaluate dietary recommendation decision engines, which can combine the existing diet scores with other variables that affect directly the tailored dietary advice, such as sex, age, BMI, lifestyle. This decision-making process is fuzzy (i.e. not simply yes or no, but similar to a percentage for each target) and some investigators are evaluating how to use fuzzy logic to evaluate diets and nutritional risks [23,24].

There are an almost infinite number of factors that can be used to increase the level of diet personalisation. These include lifestyle factors, dietary factors (e.g. food intake and preference), phenotype (e.g. body composition), and more recently genotype. The utility of such factors in influencing dietary behaviour, particularly in the context of online PN systems, remains unclear [8,25]; it is therefore vital to evaluate the efficacy of systems providing online recommendations. In order to make more progress in this field, reproducible studies, particularly randomised control trials, openly describing the decision engine for tailoring the personalised advice and evaluation of their effects on dietary change, are essential. Although the role and importance of face-to-face consultations in public health nutrition will continue, validated and effective diet apps can be part of the solution for effective dietary advice for disease prevention for the general population. For example, several weight loss studies comparing ‘usual care’ (e.g. outpatient appointments with healthcare professionals) with web-based interventions have found no significant difference between delivery methods [26,27]. The eNutri app contributes to bridging the gap between face-to face advice and that given remotely online and for this reason the decision engine has been made publicly available, to enable other researchers and organizations to conduct similar studies and contribute to its improvement [10].

## Conclusions

Evaluation of novel health-related applications by service users and expert stakeholders is vital to ensure the appropriateness, relevance and suitability of advice given. In the present study, a number of improvements to the eNutri personalised nutrition application’s diet advice were suggested by nutrition professionals including: greater participant profiling (e.g. weight and lifestyle), links to recipes and sources of further information, consideration of additional foods/nutrients of public health relevance (e.g. vitamin D), and more positive message framing/scoring. Future research will assess the efficacy of the system at improving user’s adherence to healthy eating guidance.

### Limitations

The eNutri app relies on data from a FFQ, which has some limitations. Identification of specific foods consumed is more limited compared with other methods, such as diet diaries, which may reduce its ability to identify changes in consumption of some foods. In addition, FFQs are not necessarily the nutrition assessment method that NP would utilise during nutrition consultations; 24-hour recall and food diaries are most frequently selected. This study is subjective and qualitative in nature making it more difficult to apply comparative statistics. Furthermore, the food intake on which the eNutri personalised advice was based was translated to food group equivalents by the app, however this information was not provided to the NP. In some cases, this could have contributed to the different dietary targets identified by the eNutri app and the NP. The number of female NP was much higher than male NP, which may be considered as a potential limitation. However this is reflective of the gender distribution of these professions; in the present study 16% of RNutr identified were male and in a recent international survey of RD, 4% of UK respondants were male [2].

## Supporting information

S1 File Interview protocol. (PDF)

S2 File Data input for the scenarios. (PDF)

S3 File Data output (eNutri) for the scenarios. (PDF)

S1 Table Professional nutrition recommendations according to scenarios. (PDF)

## References

[1] Franco RZ, Fallaize R, Lovegrove JA, Hwang F. Popular Nutrition-Related Mobile Apps: A Feature Assessment. JMIR mHealth uHealth. 2016;4(3):e85 10.2196/mhealth.5846 27480144 PMC4985610

[2] Chen J, Lieffers J, Bauman A, Hanning R, Allman-Farinelli M. The use of smartphone health apps and other mobile health (mHealth) technologies in dietetic practice: a three country study. J Hum Nutr Diet. 2017;30(4):439–52. 10.1111/jhn.12446 28116773

[3] Warner MM, Kelly JT, Reidlinger DP, Hoffmann TC, Campbell KL. Reporting of Telehealth-Delivered Dietary Intervention Trials in Chronic Disease: Systematic Review. J Med Internet Res. 2017;19(12):e410 10.2196/jmir.8193 29229588 PMC5742660

[4] Teasdale N, Elhussein A, Butcher F, Piernas C, Cowburn G, Hartmann-Boyce J, et al Systematic review and meta-analysis of remotely delivered interventions using self-monitoring or tailored feedback to change dietary behavior. Am J Clin Nutr. 2018;107(2):247–56. 10.1093/ajcn/nqx048 29529158 PMC5875102

[5] Celis-Morales C, Livingstone KM, Marsaux CFM, Forster H, O’Donovan CB, Woolhead C, et al Design and baseline characteristics of the Food4Me study: a web-based randomised controlled trial of personalised nutrition in seven European countries. Genes Nutr. 2015;10(1):450 10.1007/s12263-014-0450-2 25491748 PMC4261071

[6] Fallaize R, Forster H, Macready AL, Walsh MC, Mathers JC, Brennan L, et al Online Dietary Intake Estimation: Reproducibility and Validity of the Food4Me Food Frequency Questionnaire Against a 4-Day Weighed Food Record. J Med Internet Res. 2014;16(8):e190 10.2196/jmir.3355 25113936 PMC4147714

[7] Forster H, Fallaize R, Gallagher C, O’Donovan CB, Woolhead C, Walsh MC, et al Online dietary intake estimation: the Food4Me food frequency questionnaire. J Med Internet Res. 2014;16(6):e150 10.2196/jmir.3105 24911957 PMC4071230

[8] Celis-Morales C, Livingstone KM, Marsaux CFM, Macready AL, Fallaize R, O’Donovan CB, et al Effect of personalized nutrition on health-related behaviour change: evidence from the Food4me European randomized controlled trial. Int J Epidemiol. 2017; 46(2):578–588. 10.1093/ije/dyw186 27524815

[9] Zenun Franco R, Fallaize R, Lovegrove JA, Hwang F. Online dietary intake assessment using a graphical food frequency app (eNutri): Usability metrics from the EatWellUK study. PLoS One. 2018;13(8):e0202006 10.1371/journal.pone.0202006 30096211 PMC6086444

[10] Zenun Franco R.eNutri—Personalised Nutrition Web App [Internet]. 2018. Available from: 10.5281/zenodo.1304929

[11] Zenun Franco R. Online Recommender System for Personalized Nutrition Advice. In: Proceedings of the Eleventh ACM Conference on Recommender Systems—RecSys ‘17. New York, New York, USA: ACM Press; 2017. p. 411–5. Available from: http://dl.acm.org/citation.cfm?doid=3109859.3109862

[12] Chiuve SE, Fung TT, Rimm EB, Hu FB, McCullough ML, Wang M, et al Alternative dietary indices both strongly predict risk of chronic disease. J Nutr. 2012;142(6):1009–18. 10.3945/jn.111.157222 22513989 PMC3738221

[13] McCullough MML, Willett WCW. Evaluating adherence to recommended diets in adults: the Alternate Healthy Eating Index. Public Health Nutr [Internet]. 2006;9(1A):152–7. 10.1079/PHN2005938 16512963

[14] Fallaize R, Livingstone KM, Celis-Morales C, Macready AL, San-Cristobal R, Navas-Carretero S, et al Association between diet-quality scores, adiposity, total cholesterol and markers of nutritional status in european adults: Findings from the Food4Me study. Nutrients. 2018;10(1):49 10.3390/nu10010049 29316612 PMC5793277

[15] Baecke JA, Burema J, Frijters JE. A short questionnaire for the measurement of habitual physical activity in epidemiological studies. Am J Clin Nutr. 1982;36(5):936–42. 10.1093/ajcn/36.5.936 7137077

[16] Pols MA, Peeters PH, Bueno-De-Mesquita HB, Ocké MC, Wentink CA, Kemper HC, et al Validity and repeatability of a modified Baecke questionnaire on physical activity. Int J Epidemiol. 1995;24(2):381–8. 7635600 10.1093/ije/24.2.381

[17] Scientific Advisory Committee on Nutrition (SACN). Carbohydrates and health report. 2015. Available from: https://www.gov.uk/government/publications/sacn-carbohydrates-and-health-report

[18] Braun V, Clarke V. Using thematic analysis in psychology. Qual Res Psychol. 2008;3(2):77–101.

[19] Cunningham M, Wells M. Qualitative analysis of 6961 free-text comments from the first National Cancer Patient Experience Survey in Scotland. BMJ Open [Internet]. 2017;7(6):e015726 10.1136/bmjopen-2016-015726 28619780 PMC5734250

[20] Waijers PMCM, Feskens EJM, Ocke MC. A critical review of predefiend diet quality scores. Br J Nutr. 2007; 97(2):219–31. 10.1017/S0007114507250421 17298689

[21] Scientific Advisory Committee on Nutrition (SACN). Viitamin D and health report. 2016. Available from: https://www.gov.uk/government/publications/sacn-vitamin-d-and-health-report

[22] Cashman KD, Dowling KG, Škrabáková Z, Gonzalez-Gross M, Valtueña J, De Henauw S, et al Vitamin D deficiency in Europe: pandemic? Am J Clin Nutr. 2016;103(4):1033–44. 10.3945/ajcn.115.120873 26864360 PMC5527850

[23] Lee C-S, Wang M-H, Lan S-T. Adaptive Personalized Diet Linguistic Recommendation Mechanism Based on Type-2 Fuzzy Sets and Genetic Fuzzy Markup Language. IEEE Trans Fuzzy Syst. 2014;23(5):1777–802. 10.1109/TFUZZ.2014.2379256

[24] Hadianfard AM, Kareem SA, Bastani A, Karandish M. A fuzzy logic decision support system for assessing clinical nutritional risk. J Health Man Info. 2015;2(2):40

[25] Nielsen DE, El-Sohemy A. Disclosure of Genetic Information and Change in Dietary Intake: A Randomized Controlled Trial. DeAngelis MM, editor. PLoS One. 2014;9(11):e112665 10.1371/journal.pone.0112665 25398084 PMC4232422

[26] Chambliss H, Huber R, Finley CE, McDoniel SO, Kitzman-Ulrich S, et al Computerized self-monitoring and technology-assisted feedback for weight loss with and without an enhanced behavioral component.Patient Educ Couns. 2011;85(3):375–382. 10.1016/j.pec.2010.12.024 21295433

[27] McDoniel SO, Wolskee P, Shen J. Treating obesity with a novel hand-held device, computer software program, and Internet technology in primary care: the SMART motivational trial. Patient Educ Couns 2010;79(2):185–91. 10.1016/j.pec.2009.07.034 19699049

