# Supplementary material for: Evaluation of the eNutri automated personalised nutrition advice by users and nutrition professionals in the UK
Source: PLoS One. 2019 Apr 3;14(4):e0214931. doi: 10.1371/journal.pone.0214931 (PMC6447217; doi:10.1371/journal.pone.0214931)
Supplement: S1 File — (PDF) [file pone.0214931.s001.pdf]

Formative study: semi-structured interview protocol

1. Take some time to look through the report and tell me your impressions of it.
    a. Are there expressions or terms that you don’t understand?
    b. Do you have any questions about what the content means?
2. What type of food were you recommended to eat more of?
3. What type of food were you recommended to limit?
4. Does the report help you know how to change your diet to make it healthier?
5. Do you need additional to help you make changes to your diet at this moment?
6. Do you have any other comments regarding the feedback you received from the report?
