# Supplementary material for: Evaluation of the eNutri automated personalised nutrition advice by users and nutrition professionals in the UK
Source: PLoS One. 2019 Apr 3;14(4):e0214931. doi: 10.1371/journal.pone.0214931 (PMC6447217; doi:10.1371/journal.pone.0214931)
Supplement: S2 File — (PDF) [file pone.0214931.s002.pdf]

Participant 1

19-year-old male, 73kg, 1.83m, BMI 21.8kg/m^2

Analysis of Food Frequency Questionnaire, DRVs UK SACN 2015/COMA

Nutrient	Intake	Target	Limits
- ENERGY -			
**Energy (Kcal)**	**2471**	2196	
**Energy (Kj)**	**10385**	9188	
- MACRONUTRIENTS -			
**Total fat (g)**	**96**		<85
**Saturated fat (g)**	**41.4**		<26.8
**Monounsaturated fat (g)**	**33.7**	31.7	
**Polyunsaturated fat (g)**	**16.9**		>6.5 <24.4
**Long-chain Omega-3 (g)**	**0.00**		>0.49
**Protein (g)**	**110**	55	
**Carbohydrate (g)**	**325**	275	
**Sugars (g)**	**118**		N/A
**Starch (g)**	**192**		N/A
**Dietary fibre (g)**	**20.0**	30	
**Alcohol (g)**	**3.6**		<15.7
- MINERALS & TRACE ELEMENTS -			
**Sodium (mg)**	**3115**	1600	>575 <2400
**Potassium (mg)**	**3597**	3500	>2000
**Calcium (mg)**	**1437**	1000	>480
**Phosphorus (mg)**	**1779**	775	
**Magnesium (g)**	**372**	300	>190
**Iron (mg)**	**16.8**	11.3	>6.1
**Zinc (mg)**	**11.6**	9.5	>5.5
**Copper (mg)**	**1.3**	1	
**Manganese (mg)**	**3.3**		>1.2
**Selenium (µg)**	**55**	70	>40
**Iodine (µg)**	**159**	140	>70
- VITAMINS -			
**Vitamin A (µg)**	**432**	700	>300
**Vitamin D (µg)**	**3.4**	10	
**Vitamin E (mg)**	**9.4**		>4
**Thiamin (B_1) (mg)**	**2.1**	1.1	>0.23
**Riboflavin (B_2) (mg)**	**2.7**	1.3	>0.8
**Niacin (B_3) (mg)**	**34.6**	14.5	>9.7
**Pantothenoic acid (B_5) (mg)**	**6.7**	3-7	
**Vitamin B_6 (mg)**	**2.9**	1.5	>0.91
**Folate (mcg)**	**363**	200	>100
**Vitamin B_{12} (µg)**	**4.0**	1.5	>1
**Biotin (B_7) (µg)**	**53**	10-200	
**Vitamin C (mg)**	**36**	40	>10

Macronutrient Analysis

	CARBOHYDRATE	PROTEIN	FAT	ALCOHOL
Intake	325	109.8	96.2	3.6
g/kg body-weight	4.5	1.5	1.3	0.0
Kilocal %	49	17.8	35.0	1.0

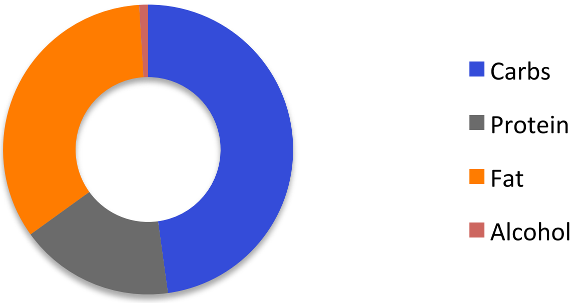

Diet Log (grams per day)

Food	Intake	Food	Intake
Breakfast non-wholegrain cereals	25.7	Nuts and seeds	2.1
Breakfast cereals	42.9	Non-creamy soups	22.9
White bread	205	Creamy sauces	21.4
Potatoes - mashed, instant, roast	20.7	Dark sauces	9.1
Potatoes - boiled, jacket	32.4	Tomato sauces	16.0
Chips	20.6	Tomato ketchup	2.6
White rice	24.7	Jam/marmalade/honey	8.1
White pasta, noodles and other grains	114	Nut or chocolate spreads	23.6
Beef, venison	11.6	Coffee, milky, latte, cappuccino	725
Stew and casserole	123	Wine	26.4
Chicken or poulty, grilled, roast	68	Beer, lager, cider	23.6
Bacon	1.6	Bananas	16.6
Sliced cold meats	33.0	Carrots	9.9
Full fat milk	189	Cabbage	6.1
High fat cheeses	40.0	Fresh/frozen peas	5.1
Egg - boiled, scrambled, omelette	24.7	Green, broad or runner beans	7.1
Salad cream, mayonnaise	14.0	Parsnips, turnips, swedes	5.1
Butter	0.6	Onions	4.4
Other vegetable oils	2.1	Garlic	1.3
Flapjacks, muesli bars, oatmeal cookies	25.7	Green salad	15.0
Chocolate snack bars	25.3	Corn	3.6
Sugar	25.0	Baked beans	13.4

Participant 2
37-year-old male, 70kg, 1.8m, BMI 21.6kg/m^2
Analysis of Food Frequency Questionnaire, DRVs UK SACN 2015/COMA

Nutrient	Intake	Target	Limits
- ENERGY -			
Energy (Kcal)	**2751**	2015	
Energy (Kj)	**11621**	8431	
- MACRONUTRIENTS -			
Total fat (g)	**103**		<78
Saturated fat (g)	**36.6**		<24.6
Monounsaturated fat (g)	**38.7**	29.1	
Polyunsaturated fat (g)	**19.4**		>6.5 <24.4
Long-chain Omega-3 (g)	**0.72**		>0.45
Protein (g)	**118**	53	
Carbohydrate (g)	**344**	252	
Sugars (g)	**147**		N/A
Starch (g)	**190**		N/A
Dietary fibre (g)	**44.6**	30	
Alcohol (g)	**2.3**		<14.4
- MINERALS & TRACE ELEMENTS -			
Sodium (mg)	**3439**	1600	>575 <2400
Potassium (mg)	**5629**	3500	>2000
Calcium (mg)	**1926**	700	>400
Phosphorus (mg)	**2124**	550	
Magnesium (g)	**467**	300	>190
Iron (mg)	**15.1**	8.7	>5.5
Zinc (mg)	**12.2**	9.5	>5.5
Copper (mg)	**1.9**	1.2	
Manganese (mg)	**6.2**		>1.4
Selenium (µg)	**78**	75	>40
Iodine (µg)	**307**	140	>70
- VITAMINS -			
Vitamin A (µg)	**707.1**	700	>300
Vitamin D (µg)	**5.6**	10	
Vitamin E (mg)	**23.0**		>4
Thiamin (B_1) (mg)	**2.6**	1	>0.23
Riboflavin (B_2) (mg)	**3.7**	1.3	>0.8
Niacin (B_3) (mg)	**24.4**	13.3	>8.9
Pantothenoic acid (B_5) (mg)	**9.5**	3-7	
Vitamin B_6 (mg)	**4.4**	1.4	>0.83
Folate (mcg)	**634**	200	>100
Vitamin B_{12} (µg)	**8.8**	1.5	>1
Biotin (B_7) (µg)	**60**	10-200	
Vitamin C (mg)	**200**	40	>10

Macronutrient Analysis

	CARBOHYDRATE	PROTEIN	FAT	ALCOHOL
Intake	344	117.6	103.4	2.3
g/kg body-weight	4.9	1.7	1.5	0.0
Kilocal %	47	17.1	33.8	0.6

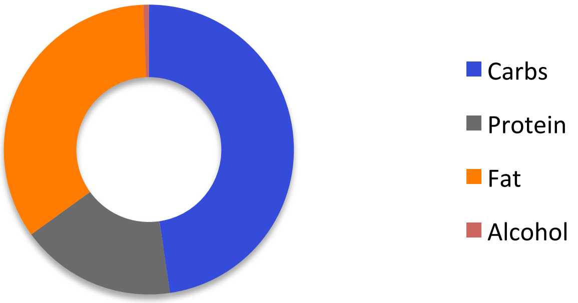

Diet Log (grams per day)

Food	Intake	Food	Intake
Porridge, Readybrek	218	Nuts and seeds	0.5
Breakfast cereals	12.1	Non-creamy soups	251
White bread	35.1	Dark sauces	7.4
Brown bread and seeded bread	5.9	Tomato sauces	90
Potatoes - mashed, instant, roast	12.8	Tomato ketchup	3.2
Potatoes - boiled, jacket	178	Jam/marmalade/honey	4.7
Brown rice, buckwheat and barley groats	92	Tea	1691
White pasta, noodles and other grains	15.9	Beer, lager, cider	47.1
Wholemeal pasta	93	Apples	147
Pizza, calzone	48.1	Pears	100
Lamb, goat	14.6	Bananas	91
Chicken or poulty, grilled, roast	43.3	Grapes	14.6
White fish	65	Carrots	29.6
Non-smoked oily fish, fresh	18.9	Butternut squash, pumpkin	4.4
Non-smoked oily fish, canned	5.4	Spinach	6.1
Smoked fish	4.9	Broccoli, spring greens, kale	66
Low-fat milk	743	Fresh/frozen peas	68
Fruit yoghurt, fruit mousse	80	Green, broad or runner beans	4.2
Low fat cheeses	13.1	Marrow, courgettes, aubergine	25.7
Milkshakes, fruit smoothies	22.6	Cauliflower	6.1
Polyunsaturated margarine	10.0	Parsnips, turnips, swedes	13.3
Olive oil	10.0	Onions	24.0
Other vegetable oils	10.0	Leeks	4.7
Plain cakes	5.4	Garlic	3.9
Flapjacks, muesli bars, oatmeal cookies	6.9	Mushrooms	18.0
Buns, muffins, pastries	10.6	Sweet peppers	11.1
Waffles, pancakes, crepes	18.6	Tomatoes	68

Chocolates, single or squares	16.0		

Participant 3

27-year-old male, 88.2kg, 1.79m, BMI 27.5kg/m^2

Analysis of Food Frequency Questionnaire, DRVs UK SACN 2015/COMA

Nutrient	Intake	Target	Limits
- ENERGY -			
**Energy (Kcal)**	**3231**	2387	
**Energy (Kj)**	**13559**	9987	
**- MACRONUTRIENTS -**			
**Total fat (g)**	**104**		<93
**Saturated fat (g)**	**42.6**		<29.2
**Monounsaturated fat (g)**	**39.6**	31.7	
**Polyunsaturated fat (g)**	**13.6**		>6.5 <26.5
**Long-chain Omega-3 (g)**	**0.00**		>0.53
**Protein (g)**	**111**	66	
**Carbohydrate (g)**	**353**	298	
**Sugars (g)**	**175**		N/A
**Starch (g)**	**155**		N/A
**Dietary fibre (g)**	**24.7**	30	
**Alcohol (g)**	**78.2**		<17.1
**- MINERALS & TRACE ELEMENTS -**			
**Sodium (mg)**	**3979**	1600	>575 <2400
**Potassium (mg)**	**4231**	3500	>2000
**Calcium (mg)**	**1076**	700	>400
**Phosphorus (mg)**	**1827**	550	
**Magnesium (g)**	**432**	300	>190
**Iron (mg)**	**26.2**	8.7	>4.7
**Zinc (mg)**	**11.0**	9.5	>5.5
**Copper (mg)**	**2.0**	1.2	
**Manganese (mg)**	**3.4**		>1.4
**Selenium (µg)**	**60**	75	>40
**Iodine (µg)**	**154**	140	>70
**- VITAMINS -**			
**Vitamin A (µg)**	**340**	700	>300
**Vitamin D (µg)**	**3.8**	10	
**Vitamin E (mg)**	**7.7**		>4
**Thiamin (B_1) (mg)**	**2.0**	1	>0.23
**Riboflavin (B_2) (mg)**	**2.3**	1.3	>0.8
**Niacin (B_3) (mg)**	**35.4**	15.8	>10.5
**Pantothenoic acid (B_5) (mg)**	**7.4**	3-7	
**Vitamin B_6 (mg)**	**3.3**	1.4	>0.98
**Folate (mcg)**	**393**	200	>100
**Vitamin B_{12} (µg)**	**5.0**	1.5	>1
**Biotin (B_7) (µg)**	**57**	10-200	
**Vitamin C (mg)**	**149**	40	>10

Macronutrient Analysis

	CARBOHYDRATE	PROTEIN	FAT	ALCOHOL
Intake	353	111.5	103.5	78.2
g/kg body-weight	4.0	1.3	1.2	0.9
Kilocal %	41	13.8	28.8	16.9

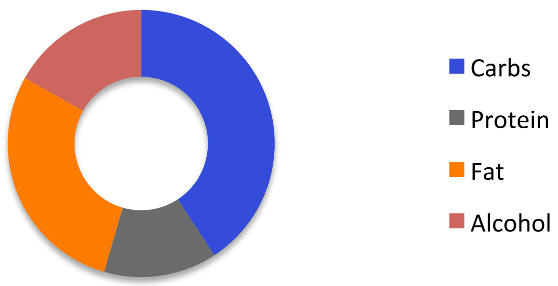

Diet Log (grams per day)

Food	Intake	Food	Intake
Breakfast wholegrain cereals	5.4	Chocolate snack bars	2.6
Breakfast non-wholegrain cereals	18.4	Sweets, toffees, mints, liquorice	1.6
White bread	60	Crisps or other packet snacks	2.1
Brown bread and seeded bread	52	Nuts and seeds	1.1
White rolls	10.3	Tomato sauces	64
Brown and seeded rolls	30.9	Tomato ketchup	1.3
Potatoes - mashed, instant, roast	20.7	Jam/marmalade/honey	0.8
Chips	10.3	Hot drinks made with milk	23.0
White rice	53.6	Hot drinks made with water	69
White pasta, noodles and other grains	25.7	Fizzy soft drinks	137
Wholemeal pasta	26.0	Pure fruit juice	47
Lasagne, moussaka, ravioli	20.5	Fruit squash/ cordial / nectar	57
Pizza, calzone	22.3	Beer, lager, cider	1556
Pork	15.7	Spirits	2.5
Stew and casserole	20.5	Apples	21.0
Burgers	18.6	Pears	29.4
Processed chicken or poultry	16.9	Oranges, satsumas, mandarins	20.0
Chicken or poultry, grilled, roast	43	Grapefruit, pineapple	7.1
Bacon	25.1	Bananas	107
Sliced cold meats	7.1	Grapes	4.3
Sausages	116	Melon, mango	9.0
Fried fish in batter	14.4	Berries	5.7
White fish	21.7	Dried fruit	1.4
Low-fat milk	81.0	Carrots	9.9
Fruit yoghurt, fruit mousse	8.9	Broccoli, spring greens, kale	9.3
Egg - boiled, scrambled, omelette	49	Fresh/frozen peas	5.1
Milkshakes, fruit smoothies	102	Onions	17.1
Butter	1.1	Garlic	3.9
Rich cakes	11.3	Sweet peppers	39
Flapjacks, muesli bars, oatmeal cookies	3.4	Tomatoes	3.6
Buns, muffins, pastries	31.7	Corn	7.1
Ice-cream, choc ices	9.1	Coleslaw, sauerkraut	7.1
Chocolates, single or squares	39.3	Baked beans	8.9

Participant 4
25-year-old male, 76kg, 1.73m, BMI 25.4kg/m^2
Analysis of Food Frequency Questionnaire, DRVs UK SACN 2015/COMA

Nutrient	Intake	Target	Limits
- ENERGY -			
Energy (Kcal)	**1771**	2196	
Energy (Kj)	**7439**	9075	
- MACRONUTRIENTS -			
Total fat (g)	**78**		<84
Saturated fat (g)	**26.4**		<26.5
Monounsaturated fat (g)	**30.5**	31.3	
Polyunsaturated fat (g)	**15.8**		>6.5 <24.1
Long-chain Omega-3 (g)	**2.71**		>0.48
Protein (g)	**106**	57	
Carbohydrate (g)	**160**	271	
Sugars (g)	**66**		N/A
Starch (g)	**90**		N/A
Dietary fibre (g)	**26.9**	30	
Alcohol (g)	**3.6**		<15.5
- MINERALS & TRACE ELEMENTS -			
Sodium (mg)	**2077**	1600	>575 <2400
Potassium (mg)	**3655**	3500	>2000
Calcium (mg)	**1171**	700	>480
Phosphorus (mg)	**1642**	550	
Magnesium (g)	**353**	300	>190
Iron (mg)	**16.5**	8.7	>4.7
Zinc (mg)	**9.9**	9.5	>5.5
Copper (mg)	**1.4**	1.2	
Manganese (mg)	**2.5**		>1.4
Selenium (µg)	**82**	75	>40
Iodine (µg)	**187**	140	>70
- VITAMINS -			
Vitamin A (µg)	**368**	700	>300
Vitamin D (µg)	**11.3**	10	
Vitamin E (mg)	**15.2**		>4
Thiamin (B_1) (mg)	**1.6**	1	>0.23
Riboflavin (B_2) (mg)	**2.6**	1.3	>0.8
Niacin (B_3) (mg)	**36.6**	14.3	>9.5
Pantothenoic acid (B_5) (mg)	**7.1**	3-7	
Vitamin B_6 (mg)	**3.3**	1.4	>0.89
Folate (mcg)	**418**	200	>100
Vitamin B_{12} (µg)	**12.5**	1.5	>1
Biotin (B_7) (µg)	**47**	10-200	
Vitamin C (mg)	**120**	40	>10

Macronutrient Analysis

	CARBOHYDRATE	PROTEIN	FAT	ALCOHOL
Intake	160	106.1	77.7	3.6
g/kg body-weight	2.1	1.4	1.0	0.0
Kilocal %	34	24.0	39.5	1.4

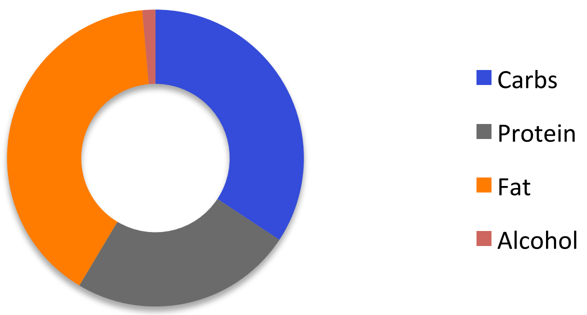

Diet Log (grams per day)

Food	Intake	Food	Intake
Breakfast wholegrain cereals	38.0	Olive oil	4.3
Breakfast cereals	35.0	Other vegetable oils	1.4
Brown bread and seeded bread	4.7	Sweet biscuits, chocolate	7.6
Tortillas, wraps	9.3	Rich cakes	4.4
Potatoes - mashed, instant, roast	7.1	Buns, muffins, pastries	12.4
Potato dishes	3.2	Nuts and seeds	12.9
Chips	7.0	Tomato sauces	64
White rice	10.6	Tea	403
Brown rice, buckwheat and barley groats	21.4	Coffee, milky, latte, cappuccino	161
Lasagne, moussaka, ravioli	53	Wine	13.2
Pizza, calzone	48	Beer, lager, cider	47
Beef, venison	8.0	Apples	10.5
Lamb, goat	7.9	Berries	51
Burgers	9.3	Dried fruit	20.0
Chicken or poulty, grilled, roast	68	Carrots	4.9
White fish	10.9	Spinach	67
Non-smoked oily fish, fresh	78	Marrow, courgettes, aubergine	4.3
Non-smoked oily fish, canned	48	Onions	40
Low-fat milk	311	Garlic	6.0
Full fat greek yoghurt	17.9	Sweet peppers	21.4
Egg - boiled, scrambled, omelette	8.1	Tomatoes	87
Butter	4.1	Corn	3.6

Participant 5
54-year-old male, 70kg, 1.74cm, BMI 23.1kg/m^2
Analysis of Food Frequency Questionnaire, DRVs UK SACN 2015/COMA

Nutrient	Intake	Target	Limits
- ENERGY -			
Energy (Kcal)	**2268**	1808	
Energy (Kj)	**9536**	7565	
- MACRONUTRIENTS -			
Total fat (g)	**89**		<70
Saturated fat (g)	**37.0**		<22.1
Monounsaturated fat (g)	**31.0**	26.1	
Polyunsaturated fat (g)	**15.2**		>6.5 <20.1
Long-chain Omega-3 (g)	**0.22**		>0.4
Protein (g)	**92**	53	
Carbohydrate (g)	**292**	226	
Sugars (g)	**110**		N/A
Starch (g)	**176**		N/A
Dietary fibre (g)	**28.9**	30	
Alcohol (g)	**3.9**		<22.6
- MINERALS & TRACE ELEMENTS -			
Sodium (mg)	**3207**	1600	>575 <2400
Potassium (mg)	**3055**	3500	>2000
Calcium (mg)	**988**	700	>480
Phosphorus (mg)	**1600**	550	
Magnesium (g)	**309**	300	>190
Iron (mg)	**15.2**	8.7	>4.7
Zinc (mg)	**10.9**	9.5	>5.5
Copper (mg)	**1.2**	1.2	
Manganese (mg)	**3.1**		>1.4
Selenium (µg)	**42**	75	>40
Iodine (µg)	**83**	140	>70
- VITAMINS -			
Vitamin A (µg)	**361**	700	>300
Vitamin D (µg)	**2.9**	10	
Vitamin E (mg)	**12.2**		>4
Thiamin (B_1) (mg)	**2.3**	0.9	>0.23
Riboflavin (B_2) (mg)	**2.3**	1.3	>0.8
Niacin (B_3) (mg)	**28.2**	11.9	>8
Pantothenoic acid (B_5) (mg)	**5.0**	3-7	
Vitamin B_6 (mg)	**3.1**	1.4	>0.75
Folate (mcg)	**442**	200	>100
Vitamin B_{12} (µg)	**4.1**	1.5	>1
Biotin (B_7) (µg)	**29**	10-200	
Vitamin C (mg)	**143**	40	>10

Macronutrient Analysis

	CARBOHYDRATE	PROTEIN	FAT	ALCOHOL
Intake	292	92.2	89.2	3.9
g/kg body-weight	4.2	1.3	1.3	0.1
Kilocal %	48	16.3	35.4	1.2

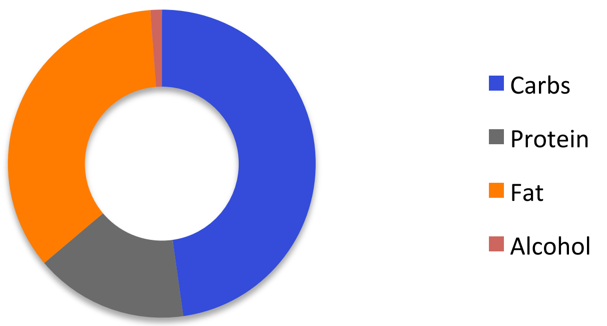

Diet Log (grams per day)

Food	Intake	Food	Intake
Breakfast cereals	100	Sponge puddings	4.9
White bread	48	Crisps or other packet snacks	4.3
Brown bread and seeded bread	53	Nuts and seeds	1.4
White rolls	3.9	Creamy soups	18.4
Cream crackers, cheese biscuits, rusks	10.3	Dark sauces	9.1
Potatoes - mashed, instant, roast	62	Tomato sauces	10.7
Potatoes - boiled, jacket	13.5	Pickles, chutney, satesaus (sate)	1.3
Chips	12.4	Marmite/bovril	4.3
White rice	24.7	Jam/marmalade/honey	1.6
White pasta, noodles and other grains	25.7	Tea	23.0
Lasagne, moussaka, ravioli	20.5	Fizzy soft drinks	46
Pizza, calzone	35.2	Pure fruit juice	140
Springrolls	7.2	Fruit squash/ cordial / nectar	37.7
Potato or Plain Dumplings	19.6	Tomato and vegetable juices	0.0
Beef, venison	8.0	Beer, lager, cider	47
Pork	7.9	Spirits	5.0
Lamb, goat	7.9	Apples	21.0
Stew and casserole	41	Oranges, satsumas, mandarins	60
Burgers	11.0	Grapes	7.3
Chicken or poulty, grilled, roast	14.4	Melon, mango	9.0
Bacon	2.3	Peaches/plums/apricots	51
Sliced cold meats	39.3	Berries	5.7
Sausages	6.9	Kiwi	6.7
Savory pies, pasties	18.3	Dried fruit	1.4
Fried fish in batter	14.4	Carrots	9.9
Non-smoked oily fish, fresh	9.4	Broccoli, spring greens, kale	9.3
Fruit yoghurt, fruit mousse	125	Brussel sprouts	12.1
High fat cheeses	30.0	Cabbage	12.3
Medium fat cheeses	3.9	Fresh/frozen peas	10.3
Egg - boiled, scrambled, omelette	8.1	Green, broad or runner beans	3.6

Quiche, savoury pancakes	12.1	Marrow, courgettes, aubergine	1.4
Low fat salad cream, mayonnaise	1.0	Cauliflower	12.3
Polyunsaturated margarine	10.0	Onions	3.0
Olive oil	0.4	Garlic	0.6
Other vegetable oils	0.4	Mushrooms	6.0
Sweet biscuits,chocolate	3.3	Sweet peppers	3.7
Sweet biscuits, plain	3.4	Beans sprouts, radish	5.0
Plain cakes	5.4	Green salad	3.6
Rich cakes	5.6	Tomatoes	12.4
Flapjacks, muesli bars, oatmeal cookies	23.0	Coleslaw, sauerkraut	3.6
Buns, muffins, pastries	5.3	Baked beans	17.9
Fruit pies, tarts, crumbles	12.1		

Participant 6
46-year-old male, 72kg, 1.83m, BMI 21.5kg/m^2
Analysis of Food Frequency Questionnaire, DRVs UK SACN 2015/COMA

Nutrient	Intake	Target	Limits
- ENERGY -			
Energy (Kcal)	**2106**	1992	
Energy (Kj)	**8831**	8335	
- MACRONUTRIENTS -			
Total fat (g)	**88**		<77
Saturated fat (g)	**22.7**		<24.3
Monounsaturated fat (g)	**33.6**	28.8	
Polyunsaturated fat (g)	**23.8**		>6.5 <22.1
Long-chain Omega-3 (g)	**0.22**		>0.44
Protein (g)	**71**	54	
Carbohydrate (g)	**246**	249	
Sugars (g)	**154**		N/A
Starch (g)	**89**		N/A
Dietary fibre (g)	**65.8**	30	
Alcohol (g)	**2.4**		<14.2
- MINERALS & TRACE ELEMENTS -			
Sodium (mg)	**1612**	1600	>575 <2400
Potassium (mg)	**5988**	3500	>2000
Calcium (mg)	**917**	700	>480
Phosphorus (mg)	**1603**	550	
Magnesium (g)	**606**	300	>190
Iron (mg)	**19.5**	8.7	>4.7
Zinc (mg)	**8.5**	9.5	>5.5
Copper (mg)	**3.1**	1.2	
Manganese (mg)	**7.4**		>1.4
Selenium (µg)	**40**	75	>40
Iodine (µg)	**100**	140	>70
- VITAMINS -			
Vitamin A (µg)	**196**	700	>300
Vitamin D (µg)	**0.9**	10	
Vitamin E (mg)	**23.0**		>4
Thiamin (B_1) (mg)	**3.4**	1	>0.23
Riboflavin (B_2) (mg)	**1.6**	1.3	>0.8
Niacin (B_3) (mg)	**20.8**	13.1	>8.5
Pantothenoic acid (B_5) (mg)	**6.3**	3-7	
Vitamin B_6 (mg)	**2.9**	1.4	>0.82
Folate (mcg)	**512**	200	>100
Vitamin B_{12} (µg)	**1.8**	1.5	>1
Biotin (B_7) (µg)	**77**	10-200	
Vitamin C (mg)	**447**	40	>10

Macronutrient Analysis

	CARBOHYDRATE	PROTEIN	FAT	ALCOHOL
Intake	246	71.2	87.8	2.4
g/kg body-weight	3.4	1.0	1.2	0.0
Kilocal %	44	13.5	37.5	0.8

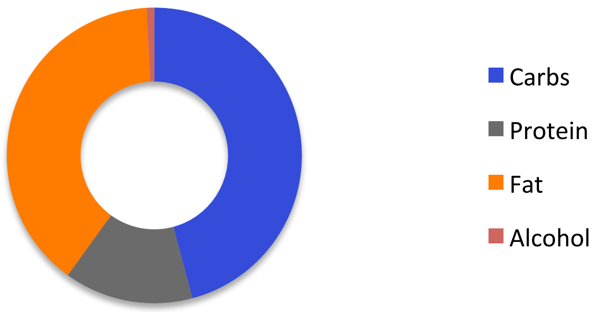

Diet Log (grams per day)

Food	Intake	Food	Intake
Porridge, Readybrek	218	Berries	298
Brown bread and seeded bread	53	Kiwi	188
Brown rice, buckwheat and barley groats	10.7	Carrots	54
Wholemeal pasta	7.9	Spinach	67
White fish	10.9	Broccoli, spring greens, kale	104
Non-smoked oily fish, fresh	9.4	Brussel sprouts	6.1
Olive oil	0.7	Cabbage	68
Sweet biscuits,chocolate	3.3	Marrow, courgettes, aubergine	2.9
Flapjacks, muesli bars, oatmeal cookies	6.9	Cauliflower	6.1
Nuts and seeds	75	Asparagus, okra	23.1
Non-creamy soups	320	Onions	40.0
Tomato sauces	118	Garlic	3.9
Nut or chocolate spreads	12.9	Mushrooms	42.0
Tea	161	Sweet peppers	3.7
Coffee, americano, black	403	Green salad	42.0
Wine	26.4	Tomatoes	87
Apples	205	Beetroot	4.2
Pears	29.4	Avocado	24.1
Oranges, satsumas, mandarins	60	Dried Lentils, beans, peas, chickpeas	116
Bananas	116	Tofu, soya meat, TVP, vegeburger	7.3
Grapes	102	Olives	2.0

Participant 7

58-year-old male, 74kg, 1.70m, BMI 25.6kg/m^2

Analysis of Food Frequency Questionnaire, DRVs UK SACN 2015/COMA

Nutrient	Intake	Target	Limits
- ENERGY -			
**Energy (Kcal)**	**2455**	1850	
**Energy (Kj)**	**10350**	7740	
**- MACRONUTRIENTS -**			
**Total fat (g)**	**82**		<72
**Saturated fat (g)**	**31.5**		<22.6
**Monounsaturated fat (g)**	**28.5**	26.7	
**Polyunsaturated fat (g)**	**15.2**		>6.5 <20.6
**Long-chain Omega-3 (g)**	**0.00**		>0.41
**Protein (g)**	**96**	56	
**Carbohydrate (g)**	**339**	231	
**Sugars (g)**	**138**		N/A
**Starch (g)**	**194**		N/A
**Dietary fibre (g)**	**30.1**	30	
**Alcohol (g)**	**3.9**		<13.2
**- MINERALS & TRACE ELEMENTS -**			
**Sodium (mg)**	**4033**	1600	>575 <2400
**Potassium (mg)**	**3552**	3500	>2000
**Calcium (mg)**	**1312**	700	>400
**Phosphorus (mg)**	**1556**	550	
**Magnesium (g)**	**359**	300	>190
**Iron (mg)**	**18.6**	8.7	>4.7
**Zinc (mg)**	**10.3**	9.5	>5.5
**Copper (mg)**	**1.4**	1.2	
**Manganese (mg)**	**5.0**		>1.4
**Selenium (µg)**	**51**	75	>40
**Iodine (µg)**	**167**	140	>70
**- VITAMINS -**			
**Vitamin A (µg)**	**554**	700	>300
**Vitamin D (µg)**	**4.6**	10	
**Vitamin E (mg)**	**14.3**		>4
**Thiamin (B_1) (mg)**	**2.6**	0.9	>0.23
**Riboflavin (B_2) (mg)**	**2.8**	1.3	>0.8
**Niacin (B_3) (mg)**	**30.3**	12.2	>8.1
**Pantothenoic acid (B_5) (mg)**	**6.3**	3-7	
**Vitamin B_6 (mg)**	**4.0**	1.4	>0.76
**Folate (mcg)**	**617**	200	>100
**Vitamin B_{12} (µg)**	**5.4**	1.5	>1
**Biotin (B_7) (µg)**	**46**	10-200	
**Vitamin C (mg)**	**131**	40	>10

Macronutrient Analysis

	CARBOHYDRATE	PROTEIN	FAT	ALCOHOL
Intake	339	95.6	81.8	3.9
g/kg body-weight	4.6	1.3	1.1	0.1
Kilocal %	52	15.6	30.0	1.1

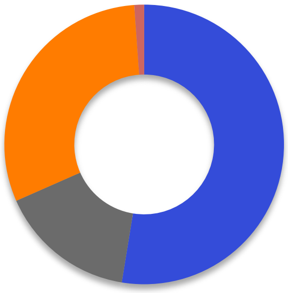

Diet Log (grams per day)

Food	Intake	Food	Intake
Breakfast non-wholegrain cereals	47.1	Nuts and seeds	1.4
White bread	9.0	Creamy soups	23.4
Brown bread and seeded bread	205	Dark sauces	12.2
Potatoes - mashed, instant, roast	25.6	Tomato sauces	12.8
Potatoes - boiled, jacket	97	Tomato ketchup	23.6
Chips	24.7	Pickles, chutney, satesaus (sate)	6.4
White rice	8.9	Jam/marmalade/honey	8.1
White pasta, noodles and other grains	15.9	Nut or chocolate spreads	12.9
Lasagne, moussaka, ravioli	20.5	Tea	725
Pizza, calzone	11.1	Coffee, milky, latte, cappuccino	69
Beef, venison	9.9	Fizzy soft drinks	137
Pork	7.9	Pure fruit juice	23.3
Lamb, goat	7.9	Fruit squash/ cordial / nectar	30.9
Processed chicken or poultry	11.9	Wine	13.2
Chicken or poulty, grilled, roast	18.6	Beer, lager, cider	23.6
Bacon	1.6	Spirits	5.0
Sliced cold meats	21.4	Apples	10.5
Sausages	6.9	Oranges, satsumas, mandarins	6.3
Pate	1.1	Bananas	91
Fried fish in batter	14.4	Grapes	4.3
White fish	21.7	Melon, mango	9.0
Shellfish, crab, prawn	3.6	Carrots	34.1
Low-fat milk	311	Spinach	1.4
High fat cheeses	12.9	Broccoli, spring greens, kale	44.6
Egg - boiled, scrambled, omelette	48.9	Cabbage	6.1
Salad cream, mayonnaise	6.0	Fresh/frozen peas	3.3
Polyunsaturated margarine	13.0	Cauliflower	60
Soft margarine	0.7	Parsnips, turnips, swedes	5.1
Other vegetable oils	0.4	Onions	0.9
Sweet biscuits,chocolate	49.3	Leeks	2.6
Flapjacks, muesli bars, oatmeal cookies	5.1	Mushrooms	2.0
Buns, muffins, pastries	5.3	Green salad	8.6

Waffles, pancakes, crepes	9.3	Tomatoes	9.8
Fruit pies, tarts, crumbles	8.3	Corn	3.6
Ice-cream, choc ices	11.7	Beetroot	4.2
Chocolate snack bars	15.9	Baked beans	8.9
Sugar	3.2		

Participant 8
52-year-old male, 71.1kg, 1.60m, BMI 27.8kg/m^2
Analysis of Food Frequency Questionnaire, DRVs UK SACN 2015/COMA

Nutrient	Intake	Target	Limits
- ENERGY -			
Energy (Kcal)	**1796**	1789	
Energy (Kj)	**7566**	7485	
- MACRONUTRIENTS -			
Total fat (g)	**67**		<70
Saturated fat (g)	**25.7**		<21.9
Monounsaturated fat (g)	**20.1**	25.8	
Polyunsaturated fat (g)	**13.0**		>6.5 <19.9
Long-chain Omega-3 (g)	**0.38**		>0.4
Protein (g)	**84**	53	
Carbohydrate (g)	**234**	224	
Sugars (g)	**152**		N/A
Starch (g)	**71**		N/A
Dietary fibre (g)	**32.2**	30	
Alcohol (g)	**9.6**		<12.8
- MINERALS & TRACE ELEMENTS -			
Sodium (mg)	**1852**	1600	>575 <2400
Potassium (mg)	**4147**	3500	>2000
Calcium (mg)	**1315**	700	>400
Phosphorus (mg)	**1571**	550	
Magnesium (g)	**352**	300	>190
Iron (mg)	**14.8**	8.7	>4.7
Zinc (mg)	**8.1**	9.5	>5.5
Copper (mg)	**1.1**	1.2	
Manganese (mg)	**2.7**		>1.4
Selenium (µg)	**37**	75	>40
Iodine (µg)	**192**	140	>70
- VITAMINS -			
Vitamin A (µg)	**303**	700	>300
Vitamin D (µg)	**2.5**	10	
Vitamin E (mg)	**13.3**		>4
Thiamin (B_1) (mg)	**1.5**	0.9	>0.23
Riboflavin (B_2) (mg)	**2.7**	1.3	>0.8
Niacin (B_3) (mg)	**25.1**	11.8	>7.9
Pantothenoic acid (B_5) (mg)	**7.1**	3-7	
Vitamin B_6 (mg)	**2.8**	1.4	>0.74
Folate (mcg)	**417**	200	>100
Vitamin B_{12} (µg)	**4.6**	1.5	>1
Biotin (B_7) (µg)	**36**	10-200	
Vitamin C (mg)	**280**	40	>10

Macronutrient Analysis

	CARBOHYDRATE	PROTEIN	FAT	ALCOHOL
Intake	234	83.7	66.9	9.6
g/kg body-weight	3.3	1.2	0.9	0.1
Kilocal %	49	18.6	33.5	3.7

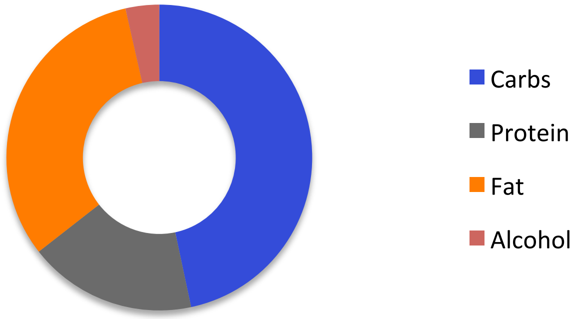

Diet Log (grams per day)

Food	Intake	Food	Intake
Porridge, Readybrek	15.1	Crisps or other packet snacks	5.7
Breakfast wholegrain cereals	38.0	Nuts and seeds	0.6
White bread	6.3	Tomato ketchup	4.3
Brown bread and seeded bread	5.9	Jam/marmalade/honey	2.7
Brown and seeded rolls	5.1	Tea	403
Tortillas, wraps	9.3	Coffee, milky, latte, cappuccino	11.5
Potatoes - mashed, instant, roast	15.3	Low calorie soft drinks	32.7
Chips	28.9	Wine	53
White rice	12.4	Beer, lager, cider	94
White pasta, noodles and other grains	19.0	Apples	116
Pizza, calzone	24.1	Pears	37.4
Beef, venison	16.0	Grapefruit, pineapple	263
Lamb, goat	14.6	Bananas	99
Chicken or poulty, grilled, roast	68	Peaches/plums/apricots	20.8
Sliced cold meats	4.2	Berries	63
Fish fingers, fish cakes	8.9	Dried fruit	3.8
Smoked fish	9.7	Carrots	12.9
Low-fat milk	189	Spinach	38.5
Low-fat natural yoghurt	98	Broccoli, spring greens, kale	14.9
High fat cheeses	8.6	Parsnips, turnips, swedes	16.3
Egg - boiled, scrambled, omelette	16.3	Onions	13.0
Low fat salad cream, mayonnaise	8.6	Garlic	0.6
French dressing, vinaigrette	13.1	Mushrooms	6.0
Milkshakes, fruit smoothies	376	Sweet peppers	29.9
Plain cakes	7.0	Green salad	19.6
Sponge puddings	7.4	Tomatoes	68
Ice-cream, choc ices	14.3	Corn	3.6
Chocolates, single or squares	10.7	Coleslaw, sauerkraut	4.7
Sweets, toffees, mints, liquorice	3.6	Baked beans	13.4

Participant 9

27-year-old female, 59.9kg, 1.68m, BMI 21.2kg/m^2

Analysis of Food Frequency Questionnaire, DRVs UK SACN 2015/COMA

Nutrient	Intake	Target	Limits
- ENERGY -			
Energy (Kcal)	**1222**	1708	
Energy (Kj)	**5146**	7146	
- MACRONUTRIENTS -			
Total fat (g)	**41**		<66
Saturated fat (g)	**17.5**		<20.9
Monounsaturated fat (g)	**14.0**	31.7	
Polyunsaturated fat (g)	**6.3**		>6.5 <19
Long-chain Omega-3 (g)	**0.10**		>0.38
Protein (g)	**42**	45	
Carbohydrate (g)	**172**	214	
Sugars (g)	**76**		N/A
Starch (g)	**94**		N/A
Dietary fibre (g)	**18.7**	30	
Alcohol (g)	**2.4**		<12.2
- MINERALS & TRACE ELEMENTS -			
Sodium (mg)	**1730**	1600	>575 <2400
Potassium (mg)	**1993**	3500	>2000
Calcium (mg)	**411**	700	>400
Phosphorus (mg)	**670**	550	
Magnesium (g)	**154**	270	>150
Iron (mg)	**6.9**	14.8	>8
Zinc (mg)	**4.9**	7	>4
Copper (mg)	**0.6**	1.2	
Manganese (mg)	**1.8**		>1.4
Selenium (µg)	**19**	60	>40
Iodine (µg)	**41**	140	>70
- VITAMINS -			
Vitamin A (µg)	**120**	600	>250
Vitamin D (µg)	**1.0**	10	
Vitamin E (mg)	**5.8**		>3
Thiamin (B_1) (mg)	**0.9**	0.8	>0.23
Riboflavin (B_2) (mg)	**0.6**	1.1	>0.8
Niacin (B_3) (mg)	**10.0**	11.3	>7.5
Pantothenoic acid (B_5) (mg)	**2.2**	3-7	
Vitamin B_6 (mg)	**1.3**	1.2	>0.7
Folate (mcg)	**185**	200	>100
Vitamin B_{12} (µg)	**1.5**	1.5	>1
Biotin (B_7) (µg)	**11**	10-200	
Vitamin C (mg)	**110**	40	>10

Macronutrient Analysis

	CARBOHYDRATE	PROTEIN	FAT	ALCOHOL
Intake	172	42.3	41.3	2.4
g/kg body-weight	2.9	0.7	0.7	0.0
Kilocal %	53	13.8	30.4	1.4

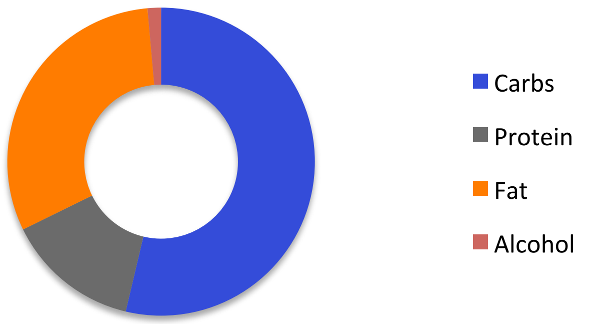

Diet Log (grams per day)

Food	Intake	Food	Intake
White bread	35.1	Egg - boiled, scrambled, omelette	12.4
Brown bread and seeded bread	35.1	Butter	6.4
Tortillas, wraps	5.9	Sweet biscuits,chocolate	45.4
Potatoes - mashed, instant, roast	92	Sugar	3.9
Potatoes - boiled, jacket	27.0	Crisps or other packet snacks	12.9
Chips	28.9	Tea	200
White rice	12.4	Low calorie soft drinks	21.4
Pizza, calzone	24.1	Fruit squash/ cordial / nectar	324
Beef, venison	11.9	Wine	26.4
Burgers	9.3	Apples	161
Bacon	3.9	Melon, mango	9.0
Sliced cold meats	28.7	Carrots	9.9
Sausages	6.9	Broccoli, spring greens, kale	44.6
Non-smoked oily fish, canned	5.4	Brussel sprouts	15.9
Full fat milk	3.5	Cauliflower	36.9
Low-fat milk	3.5	Parsnips, turnips, swedes	23.6
High fat cheeses	2.9	Baked beans	54

Participant 10
24-year-old female, 64kg, 1.65m, BMI 23.5kg/m^2
Analysis of Food Frequency Questionnaire, DRVs UK SACN 2015/COMA

Nutrient	Intake	Target	Limits
**- ENERGY -**			
**Energy (Kcal)**	**1883**	1764	
**Energy (Kj)**	**7929**	7381	
**- MACRONUTRIENTS -**			
**Total fat (g)**	**73**		<69
**Saturated fat (g)**	**20.3**		<21.6
**Monounsaturated fat (g)**	**31.7**	25.5	
**Polyunsaturated fat (g)**	**15.7**		>6.5 <19.6
**Long-chain Omega-3 (g)**	**1.09**		>0.39
**Protein (g)**	**93**	48	
**Carbohydrate (g)**	**226**	221	
**Sugars (g)**	**108**		N/A
**Starch (g)**	**106**		N/A
**Dietary fibre (g)**	**34.2**	30	
**Alcohol (g)**	**0.0**		<12.6
**- MINERALS & TRACE ELEMENTS -**			
**Sodium (mg)**	**1656**	1600	>575 <2400
**Potassium (mg)**	**4006**	3500	>2000
**Calcium (mg)**	**817**	700	>400
**Phosphorus (mg)**	**1501**	550	
**Magnesium (g)**	**338**	270	>150
**Iron (mg)**	**13.0**	14.8	>8
**Zinc (mg)**	**8.4**	7	>4
**Copper (mg)**	**1.4**	1.2	
**Manganese (mg)**	**5.1**		>1.4
**Selenium (µg)**	**76**	60	>40
**Iodine (µg)**	**176**	140	>70
**- VITAMINS -**			
**Vitamin A (µg)**	**355**	600	>250
**Vitamin D (µg)**	**6.3**	10	
**Vitamin E (mg)**	**12.6**		>3
**Thiamin (B_1) (mg)**	**1.6**	0.8	>0.23
**Riboflavin (B_2) (mg)**	**1.5**	1.1	>0.8
**Niacin (B_3) (mg)**	**24.5**	11.6	>7.8
**Pantothenoic acid (B_5) (mg)**	**6.6**	3-7	
**Vitamin B_6 (mg)**	**2.8**	1.2	>0.7
**Folate (mcg)**	**361**	200	>100
**Vitamin B_{12} (µg)**	**6.6**	1.5	>1
**Biotin (B_7) (µg)**	**50**	10-200	
**Vitamin C (mg)**	**231**	40	>10

Macronutrient Analysis

	CARBOHYDRATE	PROTEIN	FAT	ALCOHOL
Intake	226	93.4	72.5	0.0
g/kg body-weight	3.5	1.5	1.1	0.0
Kilocal %	45	19.8	34.7	0.0

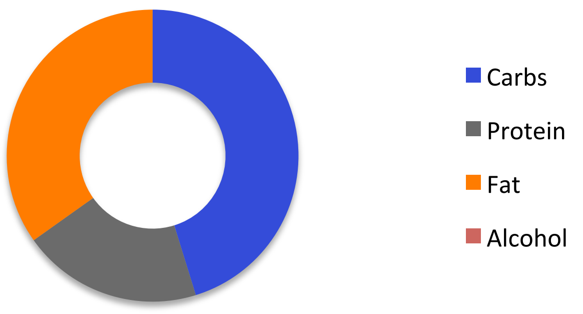

Diet Log (grams per day)

Food	Intake	Food	Intake
Porridge, Readybrek	105	Tomato sauces	8.0
Brown and seeded rolls	102	Tomato ketchup	2.1
Crispbread	30.0	Jam/marmalade/honey	8.1
Potatoes - boiled, jacket	27.0	Nut or chocolate spreads	2.1
Chips	7.0	Tea	69
White rice	21.3	Coffee, milky, latte, cappuccino	69
White pasta, noodles and other grains	10.3	Low calorie soft drinks	21.4
Wholemeal pasta	26.0	Apples	10.5
Burgers	6.1	Oranges, satsumas, mandarins	140
Chicken or poulty, grilled, roast	68	Grapes	102
Cured meats	1.6	Berries	119
Sausages	3.2	Kiwi	94
White fish	65	Dried fruit	15.5
Non-smoked oily fish, fresh	48	Carrots	45
Zero fat milk	81	Spinach	21.0
Low-fat natural yoghurt	54	Cabbage	29.4
Medium fat cheeses	3.4	Marrow, courgettes, aubergine	32.2
Egg - boiled, scrambled, omelette	49	Onions	16.5
Polyunsaturated margarine	13.0	Garlic	5.1
Olive oil	12.5	Mushrooms	2.0
Sweet biscuits, plain	34.3	Green salad	2.1
Chocolates, single or squares	2.2	Tomatoes	218
Sugar	7.5	Baked beans	26.9
Nuts and seeds	0.6		

Participant 11
25-year-old female, 97kg, 1.63m, BMI 36.5kg/m^2
Analysis of Food Frequency Questionnaire, DRVs UK SACN 2015/COMA

Nutrient	Intake	Target	Limits
- ENERGY -			
Energy (Kcal)	**2109**	2133	
Energy (Kj)	**8858**	8924	
- MACRONUTRIENTS -			
Total fat (g)	**82**		<83
Saturated fat (g)	**33.0**		<26.1
Monounsaturated fat (g)	**31.5**	31.7	
Polyunsaturated fat (g)	**12.0**		>6.5 <23.7
Long-chain Omega-3 (g)	**0.15**		>0.47
Protein (g)	**67**	73	
Carbohydrate (g)	**271**	267	
Sugars (g)	**120**		N/A
Starch (g)	**135**		N/A
Dietary fibre (g)	**14.5**	30	
Alcohol (g)	**8.3**		<15.2
- MINERALS & TRACE ELEMENTS -			
Sodium (mg)	**2243**	1600	>575 <2400
Potassium (mg)	**2213**	3500	>2000
Calcium (mg)	**694**	700	>400
Phosphorus (mg)	**1209**	550	
Magnesium (g)	**212**	270	>150
Iron (mg)	**10.8**	14.8	>8
Zinc (mg)	**7.6**	7	>4
Copper (mg)	**1.3**	1.2	
Manganese (mg)	**1.7**		>1.4
Selenium (µg)	**32**	60	>40
Iodine (µg)	**105**	140	>70
- VITAMINS -			
Vitamin A (µg)	**249**	600	>250
Vitamin D (µg)	**3.6**	10	
Vitamin E (mg)	**8.1**		>3
Thiamin (B_1) (mg)	**0.9**	0.8	>0.23
Riboflavin (B_2) (mg)	**1.1**	1.1	>0.8
Niacin (B_3) (mg)	**16.7**	14.1	>9.4
Pantothenoic acid (B_5) (mg)	**3.2**	3-7	
Vitamin B_6 (mg)	**1.5**	1.2	>0.88
Folate (mcg)	**166**	200	>100
Vitamin B_{12} (µg)	**3.4**	1.5	>1
Biotin (B_7) (µg)	**20**	10-200	
Vitamin C (mg)	**36**	40	>10

Macronutrient Analysis

	CARBOHYDRATE	PROTEIN	FAT	ALCOHOL
Intake	271	67.1	82.1	8.3
g/kg body-weight	2.8	0.7	0.8	0.1
Kilocal %	48	12.7	35.0	2.7

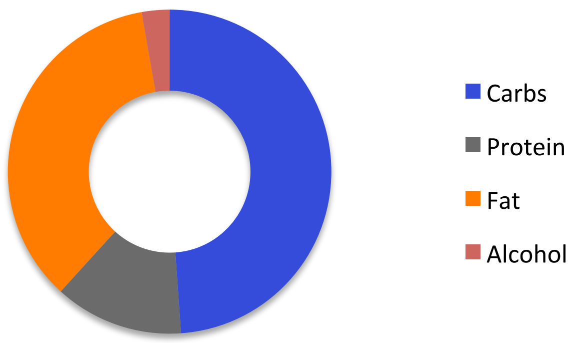

Diet Log (grams per day)

Food	Intake	Food	Intake
Breakfast non-wholegrain cereals	4.3	Sweet biscuits,chocolate	57
Breakfast cereals	6.1	Plain cakes	32.1
White bread	18.9	Rich cakes	33.9
Brown bread and seeded bread	12.0	Buns, muffins, pastries	12.4
White rolls	18.7	Ice-cream, choc ices	7.1
Tortillas, wraps	8.9	Chocolates, single or squares	3.6
Potatoes - boiled, jacket	18.9	Sugar	6.4
Chips	87	Crisps or other packet snacks	2.5
White rice	16.0	Creamy soups	23.4
White pasta, noodles and other grains	114	Dark sauces	3.7
Lasagne, moussaka, ravioli	26.6	Tomato sauces	12.8
Pizza, calzone	5.8	Tomato ketchup	1.3
Beef, venison	16.0	Jam/marmalade/honey	2.0
Burgers	18.6	Tea	95
Processed chicken or poultry	23.7	Coffee, milky, latte, cappuccino	52
Chicken or poulty, grilled, roast	22.7	Hot drinks made with milk	52
Bacon	4.6	Low calorie soft drinks	458
Sliced cold meats	7.1	Fizzy soft drinks	317
Sausages	13.7	Wine	13.2
Non-smoked oily fish, canned	7.9	Beer, lager, cider	141
Low-fat milk	41.6	Bananas	9.7
Full fat greek yoghurt	4.4	Mushrooms	4.8
High fat cheeses	17.1	Sweet peppers	3.6
Egg - boiled, scrambled, omelette	10.2	Green salad	2.1
Salad cream, mayonnaise	2.9	Tomatoes	7.1
Milkshakes, fruit smoothies	34.1	Coleslaw, sauerkraut	3.6
Soft margarine	4.3	Baked beans	17.9
Olive oil	1.4		

Participant 12
38-year-old female, 78kg, 1.65m, BMI 28.7kg/m^2
Analysis of Food Frequency Questionnaire, DRVs UK SACN 2015/COMA

Nutrient	Intake	Target	Limits
- ENERGY -			
Energy (Kcal)	**2067**	1846	
Energy (Kj)	**8697**	7724	
- MACRONUTRIENTS -			
Total fat (g)	**94**		<72
Saturated fat (g)	**39.7**		<22.6
Monounsaturated fat (g)	**34.6**	26.7	
Polyunsaturated fat (g)	**12.5**		>6.5 <20.5
Long-chain Omega-3 (g)	**2.29**		>0.41
Protein (g)	**80**	59	
Carbohydrate (g)	**220**	231	
Sugars (g)	**70**		N/A
Starch (g)	**137**		N/A
Dietary fibre (g)	**29.1**	30	
Alcohol (g)	**0.0**		<13.2
- MINERALS & TRACE ELEMENTS -			
Sodium (mg)	**1833**	1600	>575 <2400
Potassium (mg)	**3242**	3500	>2000
Calcium (mg)	**885**	700	>400
Phosphorus (mg)	**1527**	550	
Magnesium (g)	**325**	270	>150
Iron (mg)	**12.4**	14.8	>8
Zinc (mg)	**8.2**	7	>4
Copper (mg)	**1.6**	1.2	
Manganese (mg)	**5.0**		>1.4
Selenium (µg)	**74**	60	>40
Iodine (µg)	**207**	140	>70
- VITAMINS -			
Vitamin A (µg)	**601**	600	>250
Vitamin D (µg)	**7.1**	10	
Vitamin E (mg)	**9.0**		>3
Thiamin (B_1) (mg)	**1.6**	0.8	>0.23
Riboflavin (B_2) (mg)	**1.8**	1.1	>0.8
Niacin (B_3) (mg)	**19.4**	11.3	>7.5
Pantothenoic acid (B_5) (mg)	**5.0**	3-7	
Vitamin B_6 (mg)	**2.0**	1.2	>0.76
Folate (mcg)	**274**	200	>100
Vitamin B_{12} (µg)	**8.5**	1.5	>1
Biotin (B_7) (µg)	**35**	10-200	
Vitamin C (mg)	**66**	40	>10

Macronutrient Analysis

	CARBOHYDRATE	PROTEIN	FAT	ALCOHOL
Intake	220	80.2	94.4	0.0
g/kg body-weight	2.8	1.0	1.2	0.0
Kilocal %	40	15.5	41.1	0.0

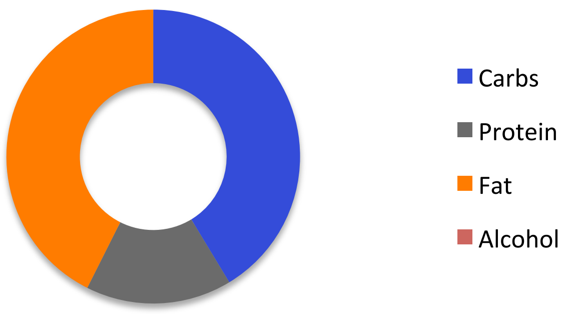

Diet Log (grams per day)

Food	Intake	Food	Intake
Porridge, Readybrek	166	Olive oil	4.3
White bread	5.9	Chocolates, single or squares	13.3
Brown bread and seeded bread	82	Nuts and seeds	3.4
Potatoes - boiled, jacket	13.5	Non-creamy soups	18.4
Chips	10.3	Tomato sauces	10.7
White rice	12.4	Marmite/bovril	2.9
Brown rice, buckwheat and barley groats	64	Jam/marmalade/honey	1.6
White pasta, noodles and other grains	77	Tea	1127
Wholemeal pasta	78	Apples	10.5
Fried fish in batter	14.4	Grapes	153
White fish	10.9	Carrots	4.9
Non-smoked oily fish, fresh	57	Broccoli, spring greens, kale	45
Non-smoked oily fish, canned	32.1	Onions	31.4
Smoked fish	9.7	Garlic	7.1
double/clotted cream	2.1	Sweet peppers	1.9
Full fat greek yoghurt	196	Green salad	1.8
High fat cheeses	2.9	Tomatoes	21.4
Egg - boiled, scrambled, omelette	16.3	Avocado	72
Salad cream, mayonnaise	6.0	Baked beans	54
Butter	37.5	Dried Lentils, beans, peas, chickpeas	50
Low fat spreads	1.4		

Participant 13

47-year-old female, 66.1kg, 1.7m, BMI 22.9kg/m^2

Analysis of Food Frequency Questionnaire, DRVs UK SACN 2015/COMA

Nutrient	Intake	Target	Limits
**- ENERGY -**			
**Energy (Kcal)**	**1926**	1669	
**Energy (Kj)**	**8079**	6983	
**- MACRONUTRIENTS -**			
**Total fat (g)**	**84**		<65
**Saturated fat (g)**	**35.8**		<20.4
**Monounsaturated fat (g)**	**29.3**	24.1	
**Polyunsaturated fat (g)**	**16.0**		>6.5 <18.5
**Long-chain Omega-3 (g)**	**2.06**		>0.37
**Protein (g)**	**116**	50	
**Carbohydrate (g)**	**129**	209	
**Sugars (g)**	**69**		N/A
**Starch (g)**	**56**		N/A
**Dietary fibre (g)**	**13.9**	30	
**Alcohol (g)**	**35.2**		<11.9
**- MINERALS & TRACE ELEMENTS -**			
**Sodium (mg)**	**3577**	1600	>575 <2400
**Potassium (mg)**	**4013**	3500	>2000
**Calcium (mg)**	**1484**	700	>400
**Phosphorus (mg)**	**1829**	550	
**Magnesium (g)**	**350**	270	>150
**Iron (mg)**	**12.4**	14.8	>8
**Zinc (mg)**	**10.2**	7	>4
**Copper (mg)**	**1.6**	1.2	
**Manganese (mg)**	**2.8**		>1.4
**Selenium (µg)**	**68**	60	>40
**Iodine (µg)**	**260**	140	>70
**- VITAMINS -**			
**Vitamin A (µg)**	**1645**	600	>250
**Vitamin D (µg)**	**6.8**	10	
**Vitamin E (mg)**	**13.0**		>3
**Thiamin (B_1) (mg)**	**1.3**	0.8	>0.23
**Riboflavin (B_2) (mg)**	**3.3**	1.1	>0.8
**Niacin (B_3) (mg)**	**30.1**	11	>7.3
**Pantothenoic acid (B_5) (mg)**	**7.6**	3-7	
**Vitamin B_6 (mg)**	**3.4**	1.2	>0.69
**Folate (mcg)**	**483**	200	>100
**Vitamin B_{12} (µg)**	**14.1**	1.5	>1
**Biotin (B_7) (µg)**	**76**	10-200	
**Vitamin C (mg)**	**86**	40	>10

Macronutrient Analysis

	CARBOHYDRATE	PROTEIN	FAT	ALCOHOL
Intake	129	116.1	83.6	35.2
g/kg body-weight	1.9	1.8	1.3	0.5
Kilocal %	25	24.1	39.0	12.8

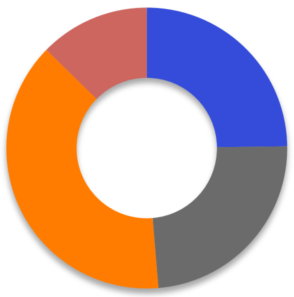

Diet Log (grams per day)

Food	Intake	Food	Intake
Brown bread and seeded bread	35.1	Flapjacks, muesli bars, oatmeal cookies	6.9
Dark wholemeal breads	4.9	Chocolates, single or squares	3.6
Cream crackers, cheese biscuits, rusks	6.9	Chocolate snack bars	2.6
Crispbread	9.4	Crisps or other packet snacks	2.1
Potatoes - mashed, instant, roast	14.1	Dark sauces	4.6
Chips	10.3	Tomato sauces	10.7
White rice	8.9	Marmite/bovril	1.4
White pasta, noodles and other grains	12.9	Jam/marmalade/honey	0.8
Pizza, calzone	24.1	Tea	403
Beef, venison	23.7	Coffee, milky, latte, cappuccino	805
Lamb, goat	14.6	Low calorie soft drinks	21.4
Chicken or poulty, grilled, roast	43.3	Wine	370
Bacon	1.6	Beer, lager, cider	23.6
Sliced cold meats	50	Apples	63
Sausages	21.1	Oranges, satsumas, mandarins	16.2
Offal	7.6	Bananas	39.9
White fish	10.9	Carrots	29.6
Non-smoked oily fish, fresh	9.4	Spinach	6.1
Non-smoked oily fish, canned	10.7	Broccoli, spring greens, kale	51
Smoked fish	42.0	Cabbage	6.1
Low-fat milk	388	Fresh/frozen peas	3.3
Low-fat natural yoghurt	54	Marrow, courgettes, aubergine	17.6
High fat cheeses	17.1	Cauliflower	6.1
Very low fat cheeses	100	Onions	9.0
Egg - boiled, scrambled, omelette	8.4	Garlic	3.9
Salad cream, mayonnaise	1.0	Mushrooms	3.0
Butter	2.1	Green salad	3.0
Low fat spreads	25.0	Tomatoes	6.2
Olive oil	4.3	Olives	2.0
Plain cakes	10.7		

Participant 14
51-year-old female, 49kg, 1.55m, BMI 20.4kg/m^2
Analysis of Food Frequency Questionnaire, DRVs UK SACN 2015/COMA

Nutrient	Intake	Target	Limits
- ENERGY -			
**Energy (Kcal)**	**2261**	1418	
**Energy (Kj)**	**9581**	5933	
- MACRONUTRIENTS -			
**Total fat (g)**	**65**		<55
**Saturated fat (g)**	**24.2**		<17.3
**Monounsaturated fat (g)**	**22.6**	20.5	
**Polyunsaturated fat (g)**	**12.6**		>6.5 <15.8
**Long-chain Omega-3 (g)**	**0.66**		>0.38
**Protein (g)**	**85**	36.8	
**Carbohydrate (g)**	**343**	177	
**Sugars (g)**	**223**		N/A
**Starch (g)**	**113**		N/A
**Dietary fibre (g)**	**46.6**	30	
**Alcohol (g)**	**7.1**		<10.1
- MINERALS & TRACE ELEMENTS -			
**Sodium (mg)**	**2516**	1600	>575 <2400
**Potassium (mg)**	**5777**	3500	>2000
**Calcium (mg)**	**1446**	700	>400
**Phosphorus (mg)**	**1799**	550	
**Magnesium (g)**	**469**	270	>150
**Iron (mg)**	**15.6**	8.7	>4.7
**Zinc (mg)**	**10.4**	7	>4
**Copper (mg)**	**1.9**	1.2	
**Manganese (mg)**	**5.4**		>1.4
**Selenium (µg)**	**64**	60	>40
**Iodine (µg)**	**232**	140	>70
- VITAMINS -			
**Vitamin A (µg)**	**520**	600	>250
**Vitamin D (µg)**	**4.6**	10	
**Vitamin E (mg)**	**12.9**		>3
**Thiamin (B_1) (mg)**	**2.5**	0.8	>0.23
**Riboflavin (B_2) (mg)**	**3.0**	1.1	>0.8
**Niacin (B_3) (mg)**	**22.0**	9.4	>6.2
**Pantothenoic acid (B_5) (mg)**	**7.9**	3-7	
**Vitamin B_6 (mg)**	**3.0**	1.2	>0.58
**Folate (mcg)**	**606**	200	>100
**Vitamin B_{12} (µg)**	**7.9**	1.5	>1
**Biotin (B_7) (µg)**	**54**	10-200	
**Vitamin C (mg)**	**412**	40	>10

Macronutrient Analysis

	CARBOHYDRATE	PROTEIN	FAT	ALCOHOL
Intake	343	85.4	65.1	7.1
g/kg body-weight	7.0	1.7	1.3	0.1
Kilocal %	57	15.1	25.9	2.2

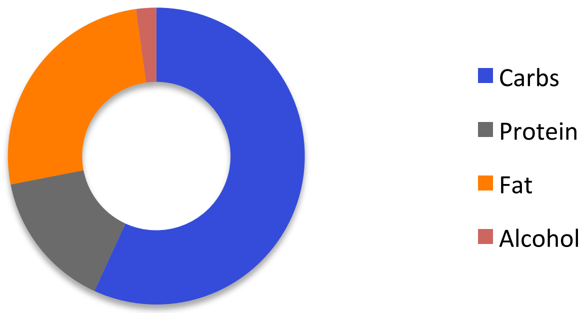

Diet Log (grams per day)

Food	Intake	Food	Intake
Porridge, Readybrek	77	Crisps or other packet snacks	1.4
Breakfast cereals	22.5	Nuts and seeds	8.0
White bread	10.9	Creamy soups	15.2
Brown bread and seeded bread	28.3	Non-creamy soups	15.1
Dark wholemeal breads	59	Creamy sauces	3.1
White rolls	2.6	Dark sauces	2.9
Brown and seeded rolls	5.1	Tomato sauces	48.0
Tortillas, wraps	2.9	Tomato ketchup	1.1
Cream crackers, cheese biscuits, rusks	6.9	Pickles, chutney, satesaus (sate)	1.7
Crispbread	9.4	Marmite/bovril	4.3
Potatoes - mashed, instant, roast	7.1	Jam/marmalade/honey	3.2
Potatoes - boiled, jacket	9.4	Tea	1127
Chips	7.0	Coffee, milky, latte, cappuccino	11.5
White rice	17.9	Pure fruit juice	261
Brown rice, buckwheat and barley groats	6.1	Fruit squash/ cordial / nectar	9.9
White pasta, noodles and other grains	15.4	Tomato and vegetable juices	18.6
Wholemeal pasta	0.0	Beer, lager, cider	141
Lasagne, moussaka, ravioli	13.6	Apples	147
Pizza, calzone	8.5	Pears	88
Springrolls	4.9	Oranges, satsumas, mandarins	459
Fish fingers, fish cakes	4.5	Grapefruit, pineapple	13.8
Fish dishes	12.4	Bananas	116
White fish	21.7	Grapes	150
Non-smoked oily fish, fresh	15.9	Melon, mango	9.0
Non-smoked oily fish, canned	8.7	Peaches/plums/apricots	8.4
Smoked fish	3.4	Berries	5.7
Shellfish, crab, prawn	21.9	Tinned, stewed fruit	7.7
Zero fat milk	340	Dried fruit	20.0
Single/sour cream	1.1	Carrots	54
Full fat greek yoghurt	4.4	Butternut squash, pumpkin	26.6
Low-fat natural yoghurt	4.4	Spinach	21.0

Fruit yoghurt, fruit mousse	98	Broccoli, spring greens, kale	27.9
High fat cheeses	8.6	Cabbage	36.9
Medium fat cheeses	1.4	Fresh/frozen peas	19.7
Low fat cheeses	4.6	Green, broad or runner beans	7.1
Very low fat cheeses	7.1	Marrow, courgettes, aubergine	17.6
Quiche, savoury pancakes	7.5	Cauliflower	6.1
Low fat salad cream, mayonnaise	0.7	Parsnips, turnips, swedes	5.1
Other salad dressing	3.0	Asparagus, okra	23.1
Milkshakes, fruit smoothies	18.1	Onions	21.0
Butter	0.6	Leeks	21.4
Polyunsaturated margarine	2.1	Garlic	1.7
Olive oil	2.1	Mushrooms	18.0
Other vegetable oils	2.1	Sweet peppers	11.1
Sweet biscuits,chocolate	0.8	Beans sprouts, radish	2.5
Sweet biscuits, plain	0.6	Green salad	19.6
Plain cakes	5.9	Tomatoes	39.3
Flapjacks, muesli bars, oatmeal cookies	1.6	Corn	3.6
Buns, muffins, pastries	3.9	Beetroot	3.5
Fruit pies, tarts, crumbles	3.8	Avocado	11.0
Milk puddings	7.0	Baked beans	4.5
Chocolates, single or squares	1.1	Dried Lentils, beans, peas, chickpeas	8.9
Sweets, toffees, mints, liquorice	0.5	Olives	1.0

Participant 15

44-year-old female, 100.8kg, 1.65m, BMI 37.0kg/m^2

Analysis of Food Frequency Questionnaire, DRVs UK SACN 2015/COMA

Nutrient	Intake	Target	Limits
- ENERGY -			
Energy (Kcal)	**1698**	2076	
Energy (Kj)	**7137**	8686	
- MACRONUTRIENTS -			
Total fat (g)	**50**		<81
Saturated fat (g)	**21.9**		<25.4
Monounsaturated fat (g)	**15.5**	30.0	
Polyunsaturated fat (g)	**8.0**		>6.5 <23.1
Long-chain Omega-3 (g)	**0.00**		>0.46
Protein (g)	**83**	76	
Carbohydrate (g)	**234**	260	
Sugars (g)	**106**		N/A
Starch (g)	**123**		N/A
Dietary fibre (g)	**20.7**	30	
Alcohol (g)	**0.0**		<14.8
- MINERALS & TRACE ELEMENTS -			
Sodium (mg)	**2822**	1600	>575 <2400
Potassium (mg)	**3556**	3500	>2000
Calcium (mg)	**1823**	700	>400
Phosphorus (mg)	**1757**	550	
Magnesium (g)	**325**	270	>150
Iron (mg)	**8.3**	14.8	>8
Zinc (mg)	**10.5**	7	>4
Copper (mg)	**0.7**	1.2	
Manganese (mg)	**3.3**		>1.4
Selenium (µg)	**32**	60	>40
Iodine (µg)	**321**	140	>70
- VITAMINS -			
Vitamin A (µg)	**952**	600	>250
Vitamin D (µg)	**7.0**	10	
Vitamin E (mg)	**4.5**		>3
Thiamin (B_1) (mg)	**1.4**	0.8	>0.23
Riboflavin (B_2) (mg)	**2.6**	1.1	>0.8
Niacin (B_3) (mg)	**16.4**	13.7	>9.1
Pantothenoic acid (B_5) (mg)	**7.3**	3-7	
Vitamin B_6 (mg)	**1.6**	1.2	>0.86
Folate (mcg)	**294**	200	>100
Vitamin B_{12} (µg)	**6.1**	1.5	>1
Biotin (B_7) (µg)	**48**	10-200	
Vitamin C (mg)	**77**	40	>10

Macronutrient Analysis

	CARBOHYDRATE	PROTEIN	FAT	ALCOHOL
Intake	234	83.0	49.9	0.0
g/kg body-weight	2.3	0.8	0.5	0.0
Kilocal %	52	19.6	26.4	0.0

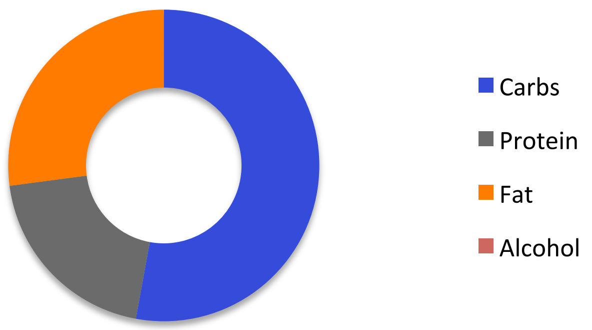

Diet Log (grams per day)

Food	Intake	Food	Intake
White bread	2.9	Sponge puddings	4.9
Brown bread and seeded bread	205	Ice-cream, choc ices	9.1
Crispbread	0.8	Chocolates, single or squares	2.9
Potatoes - mashed, instant, roast	10.4	Sweets, toffees, mints, liquorice	3.6
Chips	10.3	Crisps or other packet snacks	23.6
Springrolls	7.2	Creamy soups	18.4
Potato or Plain Dumplings	19.6	Non-creamy soups	124
Beef, venison	9.9	Dark sauces	4.6
Pork	7.9	Tomato ketchup	2.1
Stew and casserole	24.4	Jam/marmalade/honey	11.0
Burgers	6.1	Tea	17.2
Processed chicken or poultry	11.9	Coffee, americano, black	1087
Chicken or poulty, grilled, roast	7.2	Low calorie soft drinks	162
Sliced cold meats	3.6	Pure fruit juice	140
Fried fish in batter	15.9	Fruit squash/ cordial / nectar	8.6
Zero fat milk	945	Apples	10.5
Fruit yoghurt, fruit mousse	17.9	Melon, mango	14.0
High fat cheeses	12.9	Dried fruit	2.6
Low fat salad cream, mayonnaise	0.7	Carrots	5.7
Other salad dressing	0.5	Parsnips, turnips, swedes	5.1
Butter	9.0	Onions	2.2
Sweet biscuits,chocolate	9.9	Leeks	3.6
Plain cakes	4.1	Green salad	1.4
Rich cakes	4.4	Tomatoes	4.9
Flapjacks, muesli bars, oatmeal cookies	1.6	Baked beans	8.9
Buns, muffins, pastries	5.3		

Participant 16

55-year-old female, 67kg, 1.6m, BMI 26.2kg/m^2

Analysis of Food Frequency Questionnaire, DRVs UK SACN 2015/COMA

Nutrient	Intake	Target	Limits
- ENERGY -			
Energy (Kcal)	**1725**	1613	
Energy (Kj)	**7280**	6749	
- MACRONUTRIENTS -			
Total fat (g)	**50**		<63
Saturated fat (g)	**15.8**		<19.7
Monounsaturated fat (g)	**19.4**	23.3	
Polyunsaturated fat (g)	**10.7**		>6.5 <17.9
Long-chain Omega-3 (g)	**0.81**		>0.36
Protein (g)	**62**	50	
Carbohydrate (g)	**273**	202	
Sugars (g)	**135**		N/A
Starch (g)	**130**		N/A
Dietary fibre (g)	**45.6**	30	
Alcohol (g)	**1.2**		<11.5
- MINERALS & TRACE ELEMENTS -			
Sodium (mg)	**1358**	1600	>575 <2400
Potassium (mg)	**4792**	3500	>2000
Calcium (mg)	**979**	700	>400
Phosphorus (mg)	**1463**	550	
Magnesium (g)	**418**	270	>150
Iron (mg)	**16.0**	8.7	>4.7
Zinc (mg)	**8.7**	7	>4
Copper (mg)	**1.4**	1.2	
Manganese (mg)	**5.8**		>1.4
Selenium (µg)	**39**	60	>40
Iodine (µg)	**165**	140	>70
- VITAMINS -			
Vitamin A (µg)	**272**	600	>250
Vitamin D (µg)	**4.7**	10	
Vitamin E (mg)	**10.9**		>3
Thiamin (B_1) (mg)	**1.8**	0.8	>0.23
Riboflavin (B_2) (mg)	**2.0**	1.1	>0.8
Niacin (B_3) (mg)	**19.5**	10.6	>7.1
Pantothenoic acid (B_5) (mg)	**6.7**	3-7	
Vitamin B_6 (mg)	**2.8**	1.2	>0.67
Folate (mcg)	**423**	200	>100
Vitamin B_{12} (µg)	**5.4**	1.5	>1
Biotin (B_7) (µg)	**51**	10-200	
Vitamin C (mg)	**241**	40	>10

Macronutrient Analysis

	CARBOHYDRATE	PROTEIN	FAT	ALCOHOL
Intake	273	62.2	49.6	1.2
g/kg body-weight	4.1	0.9	0.7	0.0
Kilocal %	59	14.4	25.9	0.5

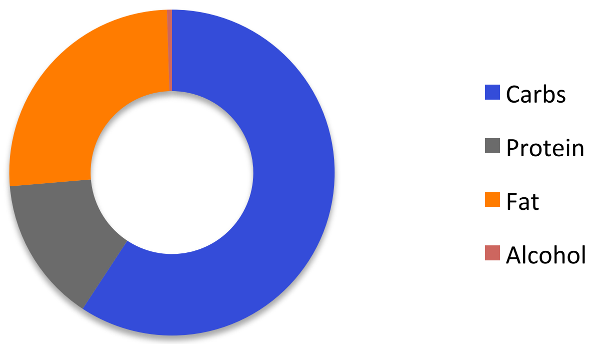

Diet Log (grams per day)

Food	Intake	Food	Intake
Porridge, Readybrek	211	Pears	88
Breakfast wholegrain cereals	27.0	Bananas	116
Breakfast cereals	7.5	Grapes	7.3
Brown bread and seeded bread	28.3	Melon, mango	28.0
Dark wholemeal breads	49.7	Peaches/plums/apricots	51
Crispbread	8.6	Berries	51
Potatoes - boiled, jacket	81	Kiwi	40.3
White rice	17.9	Dried fruit	4.7
Brown rice, buckwheat and barley groats	36.4	Carrots	54
White pasta, noodles and other grains	46.3	Butternut squash, pumpkin	5.5
Non-smoked oily fish, fresh	12.9	Spinach	3.5
Non-smoked oily fish, canned	20.1	Broccoli, spring greens, kale	36.2
Smoked fish	3.4	Brussel sprouts	12.1
Zero fat milk	146	Green, broad or runner beans	25.5
Medium fat cheeses	1.7	Marrow, courgettes, aubergine	39.7
Egg - boiled, scrambled, omelette	25.3	Cauliflower	8.0
Olive oil	5.0	Parsnips, turnips, swedes	39.9
Flapjacks, muesli bars, oatmeal cookies	1.6	Onions	24.0
Buns, muffins, pastries	3.9	Leeks	39.3
Nuts and seeds	2.8	Garlic	5.1
Non-creamy soups	90	Sweet peppers	11.1
Jam/marmalade/honey	8.6	Green salad	25.0
Tea	69	Tomatoes	69
Coffee, milky, latte, cappuccino	403	Beetroot	49.5
Pure fruit juice	98	Avocado	38.5
Wine	13.2	Olives	0.7
Apples	176		
