# Supplementary material for: Evaluation of the eNutri automated personalised nutrition advice by users and nutrition professionals in the UK
Source: PLoS One. 2019 Apr 3;14(4):e0214931. doi: 10.1371/journal.pone.0214931 (PMC6447217; doi:10.1371/journal.pone.0214931)
Supplement: S3 File — (PDF) [file pone.0214931.s003.pdf]

Participant 1, 19 years old, male, BMI = 21.8

PROVIDE FEEDBACK ADVICE SELECT PARTICIPANT ID

Hi Participant 1,

This is your personalised report.

The following messages present the most important diet changes recommended for you.

↓ RED AND PROCESSED MEAT

Your intake is VERY HIGH

- You are eating high amounts of red and processed meats.

- Based on your questionnaire, these are the food items that contributed the most to this component:

1. Sliced cold meats (ham, turkey)
2. Stew and casserole (meat and veg)
3. Beef, venison (roast, steak, mince)
4. Bacon

- Try to swap red meat with skinless chicken breast, oily fish such as salmon, turkey or legumes as they are all sources of lean protein. Swap processed meats, such as ham, with low-fat cheese in your sandwiches.

- Red and processed meats are high in saturated fat and have been linked to the development of heart disease, type 2 diabetes and some cancers.

↑ OILY FISH

Your intake is VERY LOW

- The amount of oily fish in your diet is low.

- Try to include a portion of oily fish, such as a salmon or fresh tuna, the size of a deck of cards in your weekly diet (e.g. instead of red meat for dinner) to ensure that you are meeting the minimum requirements.

- Adding more sources of oily fish into your diet will help to maintain healthy blood fat levels and prevent heart disease.

'

↑ FRUITS

Your intake is VERY LOW

- You are not eating enough fruit.

- Add more fruits into your diet by having them as a snack (e.g. apples, pears, oranges, bananas, a handful of dried fruit) or with your meals, for example by having 3-4 dates with lunch or a cup of berries with breakfast.

- Diets that are high in vitamin-rich fruit may help improve your immune system, and may help to prevent the development of certain cancers and heart diseases.

'

This section presents a summary of your progress. For each component, you receive a score between 0 and 100. Foods are divided into two groups: (1) recommended foods (the more the better, and (2) foods to limit (less is better). Your overall Healthy Eating Score is a combination of your scores from these two groups.

Healthy Eating Score

SCORE | DATE

36%

Recommended Foods

VEGETABLES | DATE

22%

FRUITS

6%

WHOLE GRAINS

9%

VEGETABLES | DATE

DAIRY PRODUCTS

100%

NUTS AND LEGUMES

52%

HEALTHY FATS

52%

OILY FISH

0%

Foods to Limit

SUGARS | DATE

84%

RED AND PROCESSED MEAT

100%

SALT

61%

ALCOHOL

0%

Participant 2, 37 years old, male, BMI = 21.6

PROVIDE FEEDBACK | ADVICE | SELECT PARTICIPANT ID

Hi Participant 2,

This is your personalised report.

The following messages present the most important diet changes recommended for you.

↑ NUTS AND LEGUMES

Your intake is VERY LOW

- You are not eating enough nuts and legumes.

- Try to have more nuts and legumes in your diet by adding a handful of nuts to your cereal, swapping a cup of rice with a cup of lentils or chickpeas, or adding seeds to your salad.

- Nuts and legumes are rich in plant-based proteins and a great source of fibre, vitamins and minerals which can lower the risk of heart disease.

↓ SALT

Your intake is VERY HIGH

- Your diet is very high in salt.

- Based on your questionnaire, these are the food items that contributed the most to this component:

1. Low fat milk (semi-skimmed milk)
2. Non-creamy soups (minestrone, vegetable)
3. Tomato sauces (in Bolognese, on meatballs or pasta)
4. Pizza, calzone
5. Dark sauces (gravy, stir-fry sauces)

- Try to limit your intake of high salt foods, such as dark sauces, bacon, smoked fish, sausages and cured meats. Instead, opt for leaner, unprocessed alternatives such as chicken breast. Avoid adding salt at the table.

- A low salt intake has been shown to benefit heart health as high amounts have been linked with the development of high blood pressure and kidney disease.

↑ HEALTHY FATS

Your intake is MEDIUM

- Good job! You are including some healthy fats in your diet but would benefit from more.

- Increase your healthy fat intake by swapping butter with liquid plant oils, and by choosing lean cuts of meat. Try having a handful of nuts each day as a snack.

- Healthy fats may help lower blood cholesterol and the chances of developing heart disease and strokes. Healthy fats are also a great source of vitamin E.

This section presents a summary of your progress. For each component, you receive a score between 0 and 100. Foods are divided into two groups: (1) recommended foods (the more the better, and (2) foods to limit (less is better). Your overall Healthy Eating Score is a combination of your scores from these two groups.

Healthy Eating Score

SCORE | DATE

75%

Recommended Foods

VEGETABLES | DATE

100%

FRUITS

100%

WHOLE GRAINS

100%

VEGETABLES DATE

DAIRY PRODUCTS

100%

NUTS AND LEGUMES

3%

HEALTHY FATS

54%

OILY FISH

100%

Foods to Limit

SUGARS DATE

24%

RED AND PROCESSED MEAT

33%

SALT

73%

ALCOHOL

0%

Participant 3, 27 years old, male, BMI = 27.5

PROVIDE FEEDBACK   ADVICE   SELECT PARTICIPANT ID

Hi Participant 3,

This is your personalised report.

The following messages present the most important diet changes recommended for you.

↓ALCOHOL

Your intake is VERY HIGH

- Your alcohol intake is very high.

- Based on your questionnaire, these are the food items that contributed the most to this component:

1. Beer, lager, cider
2. Spirits (Gin, brandy, whiskey, vodka)

- Try to reduce your alcohol intake, for example by having smaller amounts (e.g. a small glass of wine instead of a large) or swapping strong beers or wines for ones with a lower strength (ABV in %). Alternating between alcoholic and soft drinks will also help to keep you hydrated. It is also important to avoid binge drinking (i.e. drinking very large quantities of alcohol over a short period of time).

- Cutting down on alcohol helps to increase energy levels, avoid weight gain and improve mood, sleep and heart health.

↓RED AND PROCESSED MEAT

Your intake is VERY HIGH

- You are eating high amounts of red and processed meats.

- Based on your questionnaire, these are the food items that contributed the most to this component:

1. Sausages (pork, jadwurst)
2. Bacon

3. Burgers (beef, meatballs)
4. Pork (roast, chops)
5. Processed chicken or poultry (nuggets, goujons, fried)

- Try to swap red meat with skinless chicken breast, oily fish such as salmon, turkey or legumes as they are all sources of lean protein. Swap processed meats, such as ham, with low-fat cheese in your sandwiches.

- Red and processed meats are high in saturated fat and have been linked to the development of heart disease, type 2 diabetes and some cancers.

↑ OILY FISH

Your intake is VERY LOW

- The amount of oily fish in your diet is low.

- Try to include a portion of oily fish, such as a salmon or fresh tuna, the size of a deck of cards in your weekly diet (e.g. instead of red meat for dinner) to ensure that you are meeting the minimum requirements.

- Adding more sources of oily fish into your diet will help to maintain healthy blood fat levels and prevent heart disease.

This section presents a summary of your progress. For each component, you receive a score between 0 and 100. Foods are divided into two groups: (1) recommended foods (the more the better, and (2) foods to limit (less is better). Your overall Healthy Eating Score is a combination of your scores from these two groups.

Healthy Eating Score

SCORE	DATE
31%	

Recommended Foods

VEGETABLES	DATE
41%	

VEGETABLES DATE

FRUITS

65%

WHOLE GRAINS

68%

DAIRY PRODUCTS

99%

NUTS AND LEGUMES

4%

HEALTHY FATS

22%

OILY FISH

0%

Foods to Limit

SUGARS DATE

64%

RED AND PROCESSED MEAT

100%

SALT

92%

ALCOHOL

100%

Participant 4, 25 years old, male, BMI = 25.4

PROVIDE FEEDBACK | ADVICE | SELECT PARTICIPANT ID

Hi Participant 4,

This is your personalised report.

The following messages present the most important diet changes recommended for you.

↓ RED AND PROCESSED MEAT

Your intake is VERY HIGH

- You are eating high amounts of red and processed meats.

- Based on your questionnaire, these are the food items that contributed the most to this component:

1. Burgers (beef, meatballs)
2. Lasagne, moussaka, ravioli (tortellini, filled dumplings)
3. Beef, venison (roast, steak, mince)
4. Lamb, goat (roast, chops, souvlaki)

- Try to swap red meat with skinless chicken breast, oily fish such as salmon, turkey or legumes as they are all sources of lean protein. Swap processed meats, such as ham, with low-fat cheese in your sandwiches.

- Red and processed meats are high in saturated fat and have been linked to the development of heart disease, type 2 diabetes and some cancers.

↑ FRUITS

Your intake is MEDIUM

- Good job! Your diet contains some fruits but you would benefit from having even more.

- Adding a heaped tablespoon of dried fruit or a handful of fresh berries to your cereal and swapping a snack with a piece of fresh fruit are fun and easy ways to boost your fruit intake.

- Regular vitamin-rich fruit intake has been shown to have beneficial health effects and may lower the chances of developing several cancers and heart disease.

↑ NUTS AND LEGUMES

Your intake is MEDIUM

- Good job! You are including some nuts and legumes in your diet but would benefit from more.

- Adding a handful of seeds to your salad, a cup of legumes such as lentils to your main meals or having a handful of nuts as a snack are easy ways to boost your intake.

- Nuts and legumes are an excellent source of fibre, vitamins and minerals, which can lower the risk of heart disease.

This section presents a summary of your progress. For each component, you receive a score between 0 and 100. Foods are divided into two groups: (1) recommended foods (the more the better, and (2) foods to limit (less is better). Your overall Healthy Eating Score is a combination of your scores from these two groups.

Healthy Eating Score

SCORE | DATE

70%

Recommended Foods

VEGETABLES | DATE

72%

FRUITS

36%

WHOLE GRAINS

65%

VEGETABLES DATE

DAIRY PRODUCTS

100%

NUTS AND LEGUMES

50%

HEALTHY FATS

75%

OILY FISH

100%

Foods to Limit

SUGARS DATE

21%

RED AND PROCESSED MEAT

79%

SALT

24%

ALCOHOL

0%

≡

Participant 5, 54 years old, male, BMI = 23.1

PROVIDE FEEDBACK | ADVICE | SELECT PARTICIPANT ID

Hi Participant 5,

This is your personalised report.

The following messages present the most important diet changes recommended for you.

↓RED AND PROCESSED MEAT

Your intake is VERY HIGH

- You are eating high amounts of red and processed meats.

- Based on your questionnaire, these are the food items that contributed the most to this component:

1. Sliced cold meats (ham, turkey)
2. Burgers (beef, meatballs)
3. Beef, venison (roast, steak, mince)
4. Lamb, goat (roast, chops, souvlaki)
5. Pork (roast, chops)

- Try to swap red meat with skinless chicken breast, oily fish such as salmon, turkey or legumes as they are all sources of lean protein. Swap processed meats, such as ham, with low-fat cheese in your sandwiches.

- Red and processed meats are high in saturated fat and have been linked to the development of heart disease, type 2 diabetes and some cancers.

↑NUTS AND LEGUMES

Your intake is VERY LOW

- You are not eating enough nuts and legumes.

- Try to have more nuts and legumes in your diet by adding a handful of nuts to your cereal, swapping a cup of rice with a cup of lentils or chickpeas, or adding seeds to your salad.

- Nuts and legumes are rich in plant-based proteins and a great source of fibre, vitamins and minerals which can lower the risk of heart disease.

↑ WHOLE GRAINS

Your intake is VERY LOW

- You are not eating enough whole grains in your diet.

- Swapping white flour/bread/rice/pasta with brown and whole grain alternatives and sweetened cereals with whole grain cereals such as bran flakes are simple ways to increase your intake of whole grains.

- Whole grains are a great source of fibre, which is beneficial for digestion and may help to reduce the risk of heart disease and some cancers.

This section presents a summary of your progress. For each component, you receive a score between 0 and 100. Foods are divided into two groups: (1) recommended foods (the more the better, and (2) foods to limit (less is better). Your overall Healthy Eating Score is a combination of your scores from these two groups.

Healthy Eating Score

SCORE	DATE
45%	

Recommended Foods

VEGETABLES	DATE
33%	

FRUITS

60%

WHOLE GRAINS

21%

VEGETABLES DATE

DAIRY PRODUCTS

61%

NUTS AND LEGUMES

19%

HEALTHY FATS

51%

OILY FISH

87%

Foods to Limit

SUGARS DATE

70%

RED AND PROCESSED MEAT

100%

SALT

64%

ALCOHOL

0%

Participant 6, 46 years old, male, BMI = 21.5

PROVIDE FEEDBACK | ADVICE | SELECT PARTICIPANT ID

Hi Participant 6,

This is your personalised report.

The following messages present the most important diet changes recommended for you.

↑ DAIRY PRODUCTS

Your intake is VERY LOW

- You are not including enough dairy sources in your diet.

- Having a glass of milk with your breakfast, low-fat cheese in your sandwich or cup of yoghurt as a snack are simple ways to increase your dairy intake.

- Dairy products are an important source of calcium, protein and vitamin D, which all play a vital role in bone health.

'

↑ WHOLE GRAINS

Your intake is VERY LOW

- You are not eating enough whole grains in your diet.

- Swapping white flour/bread/rice/pasta with brown and whole grain alternatives and sweetened cereals with whole grain cereals such as bran flakes are simple ways to increase your intake of whole grains.

- Whole grains are a great source of fibre, which is beneficial for digestion and may help to reduce the risk of heart disease and some cancers.

'

↑ OILY FISH

Your intake is ADEQUATE

- Well done! You are including oily fish in your diet.

- Adding a variety of oily fish in your diet will ensure that you are meeting your requirement. Choose from smoked salmon/mackerel and non-smoked fish, such as fresh tuna and sardines.

- Oily fish helps to reduce your blood fat levels and therefore lowers your chances of developing heart disease.

This section presents a summary of your progress. For each component, you receive a score between 0 and 100. Foods are divided into two groups: (1) recommended foods (the more the better, and (2) foods to limit (less is better). Your overall Healthy Eating Score is a combination of your scores from these two groups.

Healthy Eating Score

SCORE	DATE
81%	

Recommended Foods

	DATE
VEGETABLES 100%	
FRUITS 100%	
WHOLE GRAINS 21%	
DAIRY PRODUCTS 0%	
NUTS AND LEGUMES 100%	

HEALTHY FATS

VEGETABLES DATE

100%

OILY FISH

87%

Foods to Limit

SUGARS DATE

9%

RED AND PROCESSED MEAT

0%

SALT

7%

ALCOHOL

0%

Participant 7, 58 years old, male, BMI = 25.6

PROVIDE FEEDBACK ADVICE SELECT PARTICIPANT ID

Hi Participant 7,

This is your personalised report.

The following messages present the most important diet changes recommended for you.

↑WHOLE GRAINS

Your intake is VERY LOW

- You are not eating enough whole grains in your diet.

- Swapping white flour/bread/rice/pasta with brown and whole grain alternatives and sweetened cereals with whole grain cereals such as bran flakes are simple ways to increase your intake of whole grains.

- Whole grains are a great source of fibre, which is beneficial for digestion and may help to reduce the risk of heart disease and some cancers.

↓RED AND PROCESSED MEAT

Your intake is VERY HIGH

- You are eating high amounts of red and processed meats.

- Based on your questionnaire, these are the food items that contributed the most to this component:

1. Sliced cold meats (ham, turkey)
2. Beef, venison (roast, steak, mince)
3. Lamb, goat (roast, chops, souvlaki)
4. Pork (roast, chops)
5. Processed chicken or poultry (nuggets, goujons, fried)

- Try to swap red meat with skinless chicken breast, oily fish such as salmon, turkey or legumes as they are all sources of lean protein. Swap processed meats, such as ham, with low-fat cheese in your sandwiches.

- Red and processed meats are high in saturated fat and have been linked to the development of heart disease, type 2 diabetes and some cancers.

'

↑OILY FISH

Your intake is VERY LOW

- The amount of oily fish in your diet is low.

- Try to include a portion of oily fish, such as a salmon or fresh tuna, the size of a deck of cards in your weekly diet (e.g. instead of red meat for dinner) to ensure that you are meeting the minimum requirements.

- Adding more sources of oily fish into your diet will help to maintain healthy blood fat levels and prevent heart disease.

'

This section presents a summary of your progress. For each component, you receive a score between 0 and 100. Foods are divided into two groups: (1) recommended foods (the more the better, and (2) foods to limit (less is better). Your overall Healthy Eating Score is a combination of your scores from these two groups.

Healthy Eating Score

SCORE | DATE

34%

Recommended Foods

VEGETABLES | DATE

52%

FRUITS

39%

WHOLE GRAINS

0%

VEGETABLES | DATE

DAIRY PRODUCTS

100%

NUTS AND LEGUMES

26%

HEALTHY FATS

45%

OILY FISH

0%

Foods to Limit

SUGARS | DATE

95%

RED AND PROCESSED MEAT

100%

SALT

94%

ALCOHOL

0%

Participant 8, 52 years old, male, BMI = 27.8

PROVIDE FEEDBACK ADVICE SELECT PARTICIPANT ID

Hi Participant 8,

This is your personalised report.

The following messages present the most important diet changes recommended for you.

↑ NUTS AND LEGUMES

Your intake is VERY LOW

- You are not eating enough nuts and legumes.

- Try to have more nuts and legumes in your diet by adding a handful of nuts to your cereal, swapping a cup of rice with a cup of lentils or chickpeas, or adding seeds to your salad.

- Nuts and legumes are rich in plant-based proteins and a great source of fibre, vitamins and minerals which can lower the risk of heart disease.

↓ RED AND PROCESSED MEAT

Your intake is VERY HIGH

- You are eating high amounts of red and processed meats.

- Based on your questionnaire, these are the food items that contributed the most to this component:

1. Beef, venison (roast, steak, mince)
2. Lamb, goat (roast, chops, souvlaki)
3. Sliced cold meats (ham, turkey)

- Try to swap red meat with skinless chicken breast, oily fish such as salmon, turkey or legumes as they are all sources of lean protein. Swap processed meats, such as ham, with low-fat cheese in your sandwiches.

- Red and processed meats are high in saturated fat and have been linked to the development of heart disease, type 2 diabetes and some cancers.

↑ WHOLE GRAINS

Your intake is MEDIUM

- Good job! You are including some whole grains in your diet but would benefit from having more.

- Try to include a wide variety of grains in your diet by having whole grain cereal or oats, whole grain bread instead of white bread and by swapping white rice or pasta with brown rice/pasta.

- Whole grains are high in fibre, B vitamins and minerals and have been shown to be protective against heart disease, some cancers and diabetes.

This section presents a summary of your progress. For each component, you receive a score between 0 and 100. Foods are divided into two groups: (1) recommended foods (the more the better, and (2) foods to limit (less is better). Your overall Healthy Eating Score is a combination of your scores from these two groups.

Healthy Eating Score

SCORE | DATE

67%

Recommended Foods

VEGETABLES | DATE

59%

FRUITS

100%

WHOLE GRAINS

47%

DAIRY PRODUCTS

VEGETABLES | DATE
100%

NUTS AND LEGUMES
2%

HEALTHY FATS
56%

OILY FISH
100%

Foods to Limit

SUGARS | DATE
33%

RED AND PROCESSED MEAT
82%

SALT
15%

ALCOHOL
0%

Participant 9, 27 years old, female, BMI = 21.2

PROVIDE FEEDBACK | ADVICE | SELECT PARTICIPANT ID

Hi Participant 9,

This is your personalised report.

The following messages present the most important diet changes recommended for you.

↑ WHOLE GRAINS

Your intake is VERY LOW

- You are not eating enough whole grains in your diet.

- Swapping white flour/bread/rice/pasta with brown and whole grain alternatives and sweetened cereals with whole grain cereals such as bran flakes are simple ways to increase your intake of whole grains.

- Whole grains are a great source of fibre, which is beneficial for digestion and may help to reduce the risk of heart disease and some cancers.

'

↑ NUTS AND LEGUMES

Your intake is VERY LOW

- You are not eating enough nuts and legumes.

- Try to have more nuts and legumes in your diet by adding a handful of nuts to your cereal, swapping a cup of rice with a cup of lentils or chickpeas, or adding seeds to your salad.

- Nuts and legumes are rich in plant-based proteins and a great source of fibre, vitamins and minerals which can lower the risk of heart disease.

'

↓ SUGARS

Your intake is VERY HIGH

- You are eating lots of sugary products.

- Based on your questionnaire, these are the food items that contributed the most to this component:

1. Fruit squash, cordial, nectar
2. Sweet biscuits, chocolate (digestive, cookies)
3. Sugar (added to tea, coffee, cereal)
4. Baked beans
5. White bread

- Try to have less in your diet. If you are craving something sweet, try having a banana, a handful of dried fruits or a sugar-free soft drink. These are sweet and healthier alternatives. Avoid adding sugar to hot drinks and breakfast cereals.

- Foods and drinks that are rich in sugars have been linked with the development of several diseases such as obesity, type 2 diabetes and heart disease.

'

This section presents a summary of your progress. For each component, you receive a score between 0 and 100. Foods are divided into two groups: (1) recommended foods (the more the better, and (2) foods to limit (less is better). Your overall Healthy Eating Score is a combination of your scores from these two groups.

Healthy Eating Score

SCORE | DATE

33%

Recommended Foods

VEGETABLES | DATE

39%

FRUITS

53%

WHOLE GRAINS

0%

VEGETABLES | DATE

DAIRY PRODUCTS

9%

NUTS AND LEGUMES

0%

HEALTHY FATS

33%

OILY FISH

41%

Foods to Limit

SUGARS | DATE

100%

RED AND PROCESSED MEAT

100%

SALT

11%

ALCOHOL

0%

Participant 10, 24 years old, female, BMI = 23.5

PROVIDE FEEDBACK | ADVICE | SELECT PARTICIPANT ID

Hi Participant 10,

This is your personalised report.

The following messages present the most important diet changes recommended for you.

↑ NUTS AND LEGUMES

Your intake is VERY LOW

- You are not eating enough nuts and legumes.

- Try to have more nuts and legumes in your diet by adding a handful of nuts to your cereal, swapping a cup of rice with a cup of lentils or chickpeas, or adding seeds to your salad.

- Nuts and legumes are rich in plant-based proteins and a great source of fibre, vitamins and minerals which can lower the risk of heart disease.

↓ SUGARS

Your intake is MODERATE

- Good job! You have been avoiding some sugary products.

- Based on your questionnaire, these are the food items that contributed the most to this component:

1. Sweet biscuits, plain (nice, ginger)
2. Sugar (added to tea, coffee, cereal)
3. Jam, marmalade, honey
4. Chocolates, single or squares
5. Crispbread (ryvita)

- Try to swap foods that are rich in free sugars such as donuts, cakes and sugary drinks with dried fruits, a small piece of dark chocolate, low-fat milk or a piece of fruit.

- High amounts of foods and drinks that are high in sugars may contribute to the development of obesity, type 2 diabetes and heart disease. Reducing your intake is extremely beneficial to your health.

↑ HEALTHY FATS

Your intake is ADEQUATE

- Well done! Your diet contains good sources of healthy fats.

- Replacing butter with liquid plant-based oils in your cooking is an easy way to boost your healthy fat intake.

- Healthy fats may lower cholesterol levels, the chances of developing heart disease and strokes and are a great source of essential vitamins.

This section presents a summary of your progress. For each component, you receive a score between 0 and 100. Foods are divided into two groups: (1) recommended foods (the more the better, and (2) foods to limit (less is better). Your overall Healthy Eating Score is a combination of your scores from these two groups.

Healthy Eating Score

SCORE	DATE
82%	

Recommended Foods

VEGETABLES	DATE
98%	

FRUITS

100%

WHOLE GRAINS

100%

VEGETABLES	DATE

DAIRY PRODUCTS

100%

NUTS AND LEGUMES

5%

HEALTHY FATS

69%

OILY FISH

100%

Foods to Limit

SUGARS	DATE

37%

RED AND PROCESSED MEAT

24%

SALT

8%

ALCOHOL

0%

Participant 11, 25 years old, female, BMI = 36.5

PROVIDE FEEDBACK ADVICE SELECT PARTICIPANT ID

Hi Participant 11,

This is your personalised report.

The following messages present the most important diet changes recommended for you.

↓SUGARS

Your intake is VERY HIGH

- You are eating lots of sugary products.

- Based on your questionnaire, these are the food items that contributed the most to this component:

1. Fizzy soft drinks (coca cola, lemonade)
2. Sweet biscuits, chocolate (digestive, cookies)
3. Rich cakes (chocolate, cheesecake)
4. Plain cakes (fruit, sponge, scones, gingerbread, raisinbread)
5. Sugar (added to tea, coffee, cereal)

- Try to have less in your diet. If you are craving something sweet, try having a banana, a handful of dried fruits or a sugar-free soft drink. These are sweet and healthier alternatives. Avoid adding sugar to hot drinks and breakfast cereals.

- Foods and drinks that are rich in sugars have been linked with the development of several diseases such as obesity, type 2 diabetes and heart disease.

↓RED AND PROCESSED MEAT

Your intake is VERY HIGH

- You are eating high amounts of red and processed meats.

- Based on your questionnaire, these are the food items that contributed the most to this component:

1. Burgers (beef, meatballs)

2. Beef, venison (roast, steak, mince)
3. Processed chicken or poultry (nuggets, goujons, fried)
4. Sausages (pork, jadwurst)
5. Sliced cold meats (ham, turkey)

- Try to swap red meat with skinless chicken breast, oily fish such as salmon, turkey or legumes as they are all sources of lean protein. Swap processed meats, such as ham, with low-fat cheese in your sandwiches.

- Red and processed meats are high in saturated fat and have been linked to the development of heart disease, type 2 diabetes and some cancers.

↑ NUTS AND LEGUMES

Your intake is VERY LOW

- You are not eating enough nuts and legumes.

- Try to have more nuts and legumes in your diet by adding a handful of nuts to your cereal, swapping a cup of rice with a cup of lentils or chickpeas, or adding seeds to your salad.

- Nuts and legumes are rich in plant-based proteins and a great source of fibre, vitamins and minerals which can lower the risk of heart disease.

This section presents a summary of your progress. For each component, you receive a score between 0 and 100. Foods are divided into two groups: (1) recommended foods (the more the better, and (2) foods to limit (less is better). Your overall Healthy Eating Score is a combination of your scores from these two groups.

Healthy Eating Score

SCORE | DATE

35%

Recommended Foods

VEGETABLES | DATE

11%

VEGETABLES

DATE

FRUITS

5%

WHOLE GRAINS

2%

DAIRY PRODUCTS

100%

NUTS AND LEGUMES

1%

HEALTHY FATS

39%

OILY FISH

60%

Foods to Limit

SUGARS

DATE

100%

RED AND PROCESSED MEAT

100%

SALT

30%

ALCOHOL

0%

Participant 12, 38 years old, female, BMI = 28.7

PROVIDE FEEDBACK ADVICE SELECT PARTICIPANT ID

Hi Participant 12,

This is your personalised report.

The following messages present the most important diet changes recommended for you.

↑ HEALTHY FATS

Your intake is MEDIUM

- Good job! You are including some healthy fats in your diet but would benefit from more.

- Increase your healthy fat intake by swapping butter with liquid plant oils, and by choosing lean cuts of meat. Try having a handful of nuts each day as a snack.

- Healthy fats may help lower blood cholesterol and the chances of developing heart disease and strokes. Healthy fats are also a great source of vitamin E.

'

↑ FRUITS

Your intake is MEDIUM

- Good job! Your diet contains some fruits but you would benefit from having even more.

- Adding a heaped tablespoon of dried fruit or a handful of fresh berries to your cereal and swapping a snack with a piece of fresh fruit are fun and easy ways to boost your fruit intake.

- Regular vitamin-rich fruit intake has been shown to have beneficial health effects and may lower the chances of developing several cancers and heart disease.

'

↑ DAIRY PRODUCTS

Your intake is MEDIUM

- Good job! You are including some dairy sources in your diet but would benefit from even

more.

- Low-fat yoghurt, low-fat cheese and/or a glass of milk are quick and easy snacks to include in your daily diet.

- Dairy products are important for bone health and are rich in calcium, vitamin D and protein.

This section presents a summary of your progress. For each component, you receive a score between 0 and 100. Foods are divided into two groups: (1) recommended foods (the more the better, and (2) foods to limit (less is better). Your overall Healthy Eating Score is a combination of your scores from these two groups.

Healthy Eating Score

SCORE	DATE
80%	

Recommended Foods

VEGETABLES	DATE
57%	

FRUITS

51%

WHOLE GRAINS

100%

DAIRY PRODUCTS

55%

NUTS AND LEGUMES

100%

VEGETABLES DATE

HEALTHY FATS

43%

OILY FISH

100%

Foods to Limit

SUGARS DATE

16%

RED AND PROCESSED MEAT

0%

SALT

15%

ALCOHOL

0%

Participant 13, 47 years old, female, BMI = 22.9

PROVIDE FEEDBACK | ADVICE | SELECT PARTICIPANT ID

Hi Participant 13,

This is your personalised report.

The following messages present the most important diet changes recommended for you.

↓ ALCOHOL

Your intake is VERY HIGH

- Your alcohol intake is very high.

- Based on your questionnaire, these are the food items that contributed the most to this component:

1. Wine
2. Beer, lager, cider

- Try to reduce your alcohol intake, for example by having smaller amounts (e.g. a small glass of wine instead of a large) or swapping strong beers or wines for ones with a lower strength (ABV in %). Alternating between alcoholic and soft drinks will also help to keep you hydrated. It is also important to avoid binge drinking (i.e. drinking very large quantities of alcohol over a short period of time).

- Cutting down on alcohol helps to increase energy levels, avoid weight gain and improve mood, sleep and heart health.

↑ WHOLE GRAINS

Your intake is VERY LOW

- You are not eating enough whole grains in your diet.

- Swapping white flour/bread/rice/pasta with brown and whole grain alternatives and sweetened cereals with whole grain cereals such as bran flakes are simple ways to increase your intake of whole grains.

- Whole grains are a great source of fibre, which is beneficial for digestion and may help to reduce the risk of heart disease and some cancers.

'

↑ NUTS AND LEGUMES

Your intake is VERY LOW

- You are not eating enough nuts and legumes.

- Try to have more nuts and legumes in your diet by adding a handful of nuts to your cereal, swapping a cup of rice with a cup of lentils or chickpeas, or adding seeds to your salad.

- Nuts and legumes are rich in plant-based proteins and a great source of fibre, vitamins and minerals which can lower the risk of heart disease.

'

This section presents a summary of your progress. For each component, you receive a score between 0 and 100. Foods are divided into two groups: (1) recommended foods (the more the better, and (2) foods to limit (less is better). Your overall Healthy Eating Score is a combination of your scores from these two groups.

Healthy Eating Score

SCORE	DATE
41%	

Recommended Foods

VEGETABLES	DATE
39%	

FRUITS

38%

WHOLE GRAINS

0%

VEGETABLES DATE

DAIRY PRODUCTS

100%

NUTS AND LEGUMES

0%

HEALTHY FATS

68%

OILY FISH

100%

Foods to Limit

SUGARS DATE

14%

RED AND PROCESSED MEAT

100%

SALT

78%

ALCOHOL

100%

Participant 14, 51 years old, female, BMI = 20.4

PROVIDE FEEDBACK ADVICE SELECT PARTICIPANT ID

Hi Participant 14,

This is your personalised report.

The following messages present the most important diet changes recommended for you.

↑ WHOLE GRAINS

Your intake is VERY LOW

- You are not eating enough whole grains in your diet.

- Swapping white flour/bread/rice/pasta with brown and whole grain alternatives and sweetened cereals with whole grain cereals such as bran flakes are simple ways to increase your intake of whole grains.

- Whole grains are a great source of fibre, which is beneficial for digestion and may help to reduce the risk of heart disease and some cancers.

'

↑ HEALTHY FATS

Your intake is MEDIUM

- Good job! You are including some healthy fats in your diet but would benefit from more.

- Increase your healthy fat intake by swapping butter with liquid plant oils, and by choosing lean cuts of meat. Try having a handful of nuts each day as a snack.

- Healthy fats may help lower blood cholesterol and the chances of developing heart disease and strokes. Healthy fats are also a great source of vitamin E.

'

↓ SUGARS

Your intake is MODERATE

- Good job! You have been avoiding some sugary products.

- Based on your questionnaire, these are the food items that contributed the most to this component:

1. Pure fruit juice (orange)
2. Breakfast cereals (muesli, cruesli)
3. Jam, marmalade, honey
4. Dark wholemeal breads (rye or soda)
5. Plain cakes (fruit, sponge, scones, gingerbread, raisinbread)

- Try to swap foods that are rich in free sugars such as donuts, cakes and sugary drinks with dried fruits, a small piece of dark chocolate, low-fat milk or a piece of fruit.

- High amounts of foods and drinks that are high in sugars may contribute to the development of obesity, type 2 diabetes and heart disease. Reducing your intake is extremely beneficial to your health.

'

This section presents a summary of your progress. For each component, you receive a score between 0 and 100. Foods are divided into two groups: (1) recommended foods (the more the better, and (2) foods to limit (less is better). Your overall Healthy Eating Score is a combination of your scores from these two groups.

Healthy Eating Score

SCORE | DATE

75%

Recommended Foods

VEGETABLES | DATE

100%

FRUITS

100%

WHOLE GRAINS

21%

DAIRY PRODUCTS

VEGETABLES | DATE
100%

NUTS AND LEGUMES
63%

HEALTHY FATS
38%

OILY FISH
100%

Foods to Limit

SUGARS | DATE
49%

RED AND PROCESSED MEAT
4%

SALT
39%

ALCOHOL
0%

Participant 15, 44 years old, female, BMI = 37.0

PROVIDE FEEDBACK ADVICE SELECT PARTICIPANT ID

Hi Participant 15,

This is your personalised report.

The following messages present the most important diet changes recommended for you.

↑ WHOLE GRAINS

Your intake is VERY LOW

- You are not eating enough whole grains in your diet.

- Swapping white flour/bread/rice/pasta with brown and whole grain alternatives and sweetened cereals with whole grain cereals such as bran flakes are simple ways to increase your intake of whole grains.

- Whole grains are a great source of fibre, which is beneficial for digestion and may help to reduce the risk of heart disease and some cancers.

'

↑ NUTS AND LEGUMES

Your intake is VERY LOW

- You are not eating enough nuts and legumes.

- Try to have more nuts and legumes in your diet by adding a handful of nuts to your cereal, swapping a cup of rice with a cup of lentils or chickpeas, or adding seeds to your salad.

- Nuts and legumes are rich in plant-based proteins and a great source of fibre, vitamins and minerals which can lower the risk of heart disease.

'

↑ OILY FISH

Your intake is VERY LOW

- The amount of oily fish in your diet is low.

- Try to include a portion of oily fish, such as a salmon or fresh tuna, the size of a deck of cards in your weekly diet (e.g. instead of red meat for dinner) to ensure that you are meeting the minimum requirements.

- Adding more sources of oily fish into your diet will help to maintain healthy blood fat levels and prevent heart disease.

'

This section presents a summary of your progress. For each component, you receive a score between 0 and 100. Foods are divided into two groups: (1) recommended foods (the more the better, and (2) foods to limit (less is better). Your overall Healthy Eating Score is a combination of your scores from these two groups.

Healthy Eating Score

SCORE | DATE

32%

Recommended Foods

VEGETABLES | DATE

21%

FRUITS

11%

WHOLE GRAINS

0%

DAIRY PRODUCTS

100%

NUTS AND LEGUMES

0%

VEGETABLES DATE

HEALTHY FATS

28%

OILY FISH

0%

Foods to Limit

SUGARS DATE

65%

RED AND PROCESSED MEAT

91%

SALT

50%

ALCOHOL

0%

Participant 16, 55 years old, female, BMI = 26.2

PROVIDE FEEDBACK | ADVICE | SELECT PARTICIPANT ID

Hi Participant 16,

This is your personalised report.

The following messages present the most important diet changes recommended for you.

↑ NUTS AND LEGUMES

Your intake is VERY LOW

- You are not eating enough nuts and legumes.

- Try to have more nuts and legumes in your diet by adding a handful of nuts to your cereal, swapping a cup of rice with a cup of lentils or chickpeas, or adding seeds to your salad.

- Nuts and legumes are rich in plant-based proteins and a great source of fibre, vitamins and minerals which can lower the risk of heart disease.

'

↑ HEALTHY FATS

Your intake is MEDIUM

- Good job! You are including some healthy fats in your diet but would benefit from more.

- Increase your healthy fat intake by swapping butter with liquid plant oils, and by choosing lean cuts of meat. Try having a handful of nuts each day as a snack.

- Healthy fats may help lower blood cholesterol and the chances of developing heart disease and strokes. Healthy fats are also a great source of vitamin E.

'

↓ SUGARS

Your intake is LOW

- Well done! You are avoiding high sugar products.

- Based on your questionnaire, these are the food items that contributed the most to this component:

1. Pure fruit juice (orange)
2. Jam, marmalade, honey
3. Dark wholemeal breads (rye or soda)
4. Breakfast cereals (muesli, cruesli)
5. Breakfast wholegrain cereals (branflakes, barley flakes)

- Try to reduce your intake even further for maximum health benefit by swapping sugary fizzy drinks with sparkling water and sweet desserts with a piece of fruit/dried fruit.

- Foods that are rich in sugar have been linked with the development of obesity, type 2 diabetes and heart disease.

'

This section presents a summary of your progress. For each component, you receive a score between 0 and 100. Foods are divided into two groups: (1) recommended foods (the more the better, and (2) foods to limit (less is better). Your overall Healthy Eating Score is a combination of your scores from these two groups.

Healthy Eating Score

SCORE | DATE

82%

Recommended Foods

VEGETABLES | DATE

100%

FRUITS

100%

WHOLE GRAINS

79%

DAIRY PRODUCTS

VEGETABLES DATE

100%

NUTS AND LEGUMES

11%

HEALTHY FATS

45%

OILY FISH

100%

Foods to Limit

SUGARS DATE

33%

RED AND PROCESSED MEAT

0%

SALT

0%

ALCOHOL

0%
