# Supplementary material for: Evaluation of the eNutri automated personalised nutrition advice by users and nutrition professionals in the UK
Source: PLoS One. 2019 Apr 3;14(4):e0214931. doi: 10.1371/journal.pone.0214931 (PMC6447217; doi:10.1371/journal.pone.0214931)
Supplement: S1 Table — (PDF) [file pone.0214931.s004.pdf]

S1 Table. Professional nutrition recommendations according to scenarios (n=12 responses per scenario; i.e. 3 recommendations x 4 NP) [a]

Scenario	Nutrition targets selected by nutrition professionals
1	SFA (n=3), fibre (n=4)*, sugar (n=2), salt (n=2), vitamin D (n=1)*
2	None (n=3), salt (n=2), energy (n=1), fibre (n=1)*, SFA (n=1), macronutrient ratio (n=1), vitamin D (n=1)*, fruit (n=1)*, sugar (n=1),
3	Alcohol (n=4), sodium (n=2), energy (n=1), fibre (n=1)*, fat composition (n=1), SFA (n=1), snack foods (n=1), vegetables (n=1)*
4	Fruit (n=3)*, sugar (n=2), fibre (n=2)*, low-fat dairy (n=1)*, vegetables (n=1)*, SFA (n=1), protein (n=1)*, coffee (n=1)
5	Carbohydrate composition (n=3), SFA (n=2), salt (n=2), red meat (n=1), protein (n=1), pizza (n=1), vitamin D (n=1)*, Brazil nuts (n=1)*
6	Total fat (n=2), oily fish (n=2)*, long chain omega-3 fatty acids (n=1)*, MUFA (n=1)*, vitamin A (n=1)*, starchy carbohydrate (n=2)*, fruit (n=1)*, fruit (n=1), phosphorus (n=1)
7	Energy (n=3), SFA (n=2), sugar (n=2), salt (n=2), vitamin D (n=2)*, alcohol (n=1)
8	Sugar (n=3), SFA (n=2), vitamin A (n=2)*, salt (n=1), fruit (n=1), vitamin D (n=1)*, breakfast (n=1), n/a (n=1)
9	Energy (n=2)*, fibre (n=2)*, fat composition (n=1), MUFA (n=1)*, iodine (n=1)*, vitamin A (n=1)*, calcium (n=1)*, iron (n=1)*, fish (n=1)*, sugar (n=1)
10	Vitamin D (n=3)*, iron (n=3)*, protein (n=2), fat (n=1), sugar (n=1), fluid (n=1)*, starchy carbohydrate (n=1)*
11	Fibre (n=2)*, SFA (n=2), refined carbohydrate (n=1), carbohydrate (n=1), sugar (n=1), salt (n=1), protein (n=1), selenium (n=1)*, red/processed meat (n=1), fruit (n=1)*
12	Energy (n=2), total fat (n=1), fat composition (n=1), fruit (n=1)*, omega-3 fatty acids (n=1)*, protein (n=1), carbohydrate (n=1)*, complex carbohydrate (n=1)*, folate (n=1)*, vitamin D (n=1)*, breakfast (n=1)
13	Alcohol (n=4), total fat (n=2), saturated fat (n=1), salt (n=2), fibre (n=2)*, takeaway (n=1)
14	Vitamin D (n=3)*, carbohydrate composition (n=2), fruit juice (n=2), fruit (n=1), dairy (n=1)*, SFA (n=1), salt (n=1), n/a (n=1)
15	Oily fish (n=2)*, SFA (n=1), fibre (n=1)*, sugar (n=1), high calorie snacks (n=1)*, fried food (n=1), fruit (n=1)*, vegetables (n=1)*, salt (n=1), iron (n=1)*, simple carbohydrates (n=1)
16	Vitamin D (n=2), energy (n=1), SFA (n=1), sugar (n=1), carbohydrate (n=1), vitamin A (n=1)*, selenium (n=1)*, fruit (n=1)*, n/a (n=3)

[a] * Indicates advice to increase target; n/a, nutrient or food target not identified (e.g. NP stated more information required)
